# The association of triglyceride-glucose index with cancer incidence and mortality: a systematic review and meta-analysis of cohort studies

**DOI:** 10.3389/fendo.2025.1682062

**Published:** 2025-10-24

**Authors:** Shantong Peng, Caiyu Wang, Qianxun Liu, Qi Luo

**Affiliations:** ^1^ Country Queen Mary School, Jiangxi Medical College, Nanchang University, Nanchang, Jiangxi, China; ^2^ College of Basic Medical Sciences, Nanchang University, Nanchang, Jiangxi, China

**Keywords:** cancer, triglyceride-glucose index, insulin resistance, systematic review, meta-analysis

## Abstract

**Purpose:**

This study seeks to summarize the relationship between the triglyceride-glucose (TyG) index, a novel and reliable surrogate indicator of insulin resistance, and cancer-associated outcomes through meta-analysis.

**Methods:**

A thorough search was performed in PubMed, Embase, and Web of Science to identify cohort studies examining the relationship between the TyG index and cancer-associated outcomes. Adjusted hazard ratios (HR) with their 95% confidence intervals (CI) were pooled through random-effect meta-analyses. HRs were calculated using the TyG index as a continuous variable and as a categorial variable.

**Results:**

Twenty-two studies were included in this systematic review. Meta-analysis of seven studies comparing the highest TyG group to the lowest TyG group showed a significantly increased risk of cancer in the former group (HR = 1.16, 95% CI 1.08-1.25). The same result was found when pooling the HRs from studies analyzing the TyG index as a continuous variable (HR = 1.08, 95% CI 1.05-1.11). The pooled results of two studies identified a significant association between the TyG index and cancer-related mortality among cancer-diagnosed people (HR = 1.21, 95% CI 1.06-1.39). Additionally, studies observed no association between the TyG index and cancer-related mortality among cancer-free people (HR = 1.02, 95% CI 0.93-1.12), as well as all-cause mortality in cancer patients (HR = 0.98, 95% CI 0.58-1.64).

**Conclusion:**

Our findings indicate that a higher TyG index is associated with an increased risk of cancer, but shows no significant overall association with cancer prognosis. Further investigation is essential to substantiate these associations and evaluate the clinical value of the TyG index.

**Systematic Review Registration:** https://www.crd.york.ac.uk/PROSPERO/, identifier PROSPERO (420251014091).

## 1 Introduction

Cancer is one of the leading causes of death globally, with its increasing incidence placing a significant strain on both public health and healthcare systems (1). Currently, due to the prevalence of poor diet and lifestyle habits, people are at an increasing risk of developing cancer, particularly some obesity-related cancers such as colorectal cancer, pancreatic cancer, and prostate cancer (1).

Insulin resistance (IR) is a pathophysiological condition characterized with a diminished biological response of peripheral tissues to insulin, leading to impaired glucose homeostasis. It constitutes a central mechanism underlying the development of type II diabetes mellitus (DM) and various metabolic syndromes. The triglyceride-glucose (TyG) index, derived from fasting plasma triglyceride and glucose concentrations, has emerged as a reliable and cost-effective surrogate marker for assessing IR (2). Numerous studies have demonstrated that the TyG index exhibits strong correlations with the hyperinsulinemic-euglycemic clamp technique, which is regarded as the gold standard for IR measurement (2–4). Currently, the TyG index is increasingly being utilized in epidemiological and clinical research to evaluate insulin resistance and predict metabolic-correlated disease risks (2, 5, 6).

Recently, there have been a few individual studies addressing the relationship between the TyG index and cancer occurrence or prognosis in the literature. However, discrepancies among studies exist, and the controversy between different opinions persists (7–9). This study aims to examine the correlation between the TyG index and cancer-associated outcomes, respectively, cancer incidence, cancer-related mortality among cancer-free people and cancer-diagnosed people, and all-cause mortality among cancer patients.

## 2 Methods

The protocol was registered with PROSPERO (International Prospective Register of Systematic Reviews, https://www.crd.york.ac.uk/PROSPERO/) under registration number CRD 420251014091. We reported this meta-analysis of cohort studies in accordance with the Preferred Reporting Item for Systematic Review and Meta-Analysis 2020 guidelines (PRISMA 2020) (**Supplementary Table 2**).

### 2.1 Data sources and search strategy

The PubMed, Embase and Web of Science were searched comprehensively for studies from inception to July 18, 2025. The search terms were as followed: (“TyG” or “triglyceride-glucose index” or “triacylglycerol-glucose index” or “triglyceride glucose index”) and (“cancer*” or “tumor*” or “neoplasm*” or “malignan*”). And advanced search with “Title/Abstract” as search fields was adopted. The details of the search strategy are described in **Supplementary Table 1**.

### 2.2 Study selection

We used EndNote 21 software to organize all studies. Following the removal of the duplicates, two reviewers independently screened the titles and abstracts sourced from the database and performed full-text screening to identify the available studies. Studies that meet all of the following criteria were included: (1) participants: adults (≥18 years of age) of any sex or ethnicity; (2) types of studies: cohort studies published as full-length articles; (3) outcomes of interest: cancer incidence and cancer-related death; (4) exposure and comparator: the TyG index calculated as Ln(Fasting Triglycerides [mg/dL] × [Fasting Plasma Glucose [mg/dL]/2); (5) the estimate effects were reported with multivariate analysis. Studies that meet the following criteria were excluded: (1) estimate effects were reported with univariate analysis; (2) the HR for the association between the TyG index and outcomes of interest was not reported; (3) duplicated studies were based on the same sample; (4) no relevant outcomes were reported; (5) not a cohort design. All excluded studies for reasons (n=36) are shown in **Supplementary Table 3**. Any inconsistencies during the process were settled by the third reviewer.

### 2.3 Data extraction

Two reviewers extracted the data independently from the included studies. The extracted data encompassed study characteristics (first author, country, publication year, number of participants, follow-up duration), participants characteristics (baseline age, sex ratio), TyG index analysis model (categorial or continuous), main outcome (cancer types, the assessment of outcomes), and adjusted covariates. Besides, the estimates with 95% CI of the TyG index and outcomes in individual studies were also extracted and integrated. Afterwards, the data extracted by two reviewers was exchanged for validation. Any discrepancies were solved by the third reviewer. The details of data extraction are described in **Tables 1**, **2**.

**Table 1:** Basic characteristics of included studies.

	The association between TyG index and cancer incidence (N = 9)						
ID	Author, year	Country	Participants	Age (SD)	Male (%)	Outcomes	Reference
1	Wu Z, 2025	UK	428,152	56.54 (8.09)	46.1	1,759 pancreatic cancer cases	(33)
2	Yang C, 2024	UK	388,900	57.0	47.2	779 esophageal cancer cases	(18)
3	Son M, 2024	Korea	314,141	Q1: 57.8 (8.5) Q2: 59.0 (8.8) Q3: 59.4 (8.8) Q4: 59.1 (8.7)	Q1: 45.6 Q2: 50.8 Q3: 55.5 Q4: 63.6	6,112 colorectal cancer cases	(20)
4	Kityo A, 2024	Korea	98,800	53.2 (8.3)	33.45	699 colorectal cancer cases	(21)
5	Jochems SHJ, 2023	Sweden	68,147	47.3 (10.3)	100	3,940 prostate cancer cases	(29)
6	Liu T, 2022	China	93,659	51.44 (12.45)	79.73	593 colorectal cancer cases	(17)
7	Wang L, 2021	UK	324,334	55.831 (8.051)	44.176	1,593 lung cancer cases	(30)
8	Okamura T, 2020	Japan	27,921	45.7 (10.1)	58.86	116 colorectal cancer cases	(27)
9	Fritz J, 2020	Europe	510,471	43.1 (10.6) Q1: 39.6 (11.0) Q2: 42.8 (10.7) Q3: 43.6 (10.7) Q4: 44.4 (10.2) Q5: 44.9 (9.4)	50.5 Q1: 32.3 Q2: 40.5 Q3: 48.7 Q4: 58.7 Q5: 72.5	16,052 obesity-related cancer cases	(7)

	The association between TyG index and cancer mortality (N = 16)						
ID	Author, year	Country	Participants	Age (SD)	Male (%)	Outcomes	Reference
I. The association between TyG index and cancer mortality in cancer-free people (6)							
1	Li S, 2024	US	27,642 (cancer-free)	47.4 (19.2)	50.2	914 cancer deaths	(26)
2	Fritz J, 2024	Europe	259,884 (cancer-free)	43.3 (10.1) Q1: 41.3 (10.2) Q2: 43.1 (10.3) Q3: 44.0 (10.0) Q4: 44.9 (9.5)	100	1,784 prostate cancer deaths	(22)
3	He G, 2024	China	3,524,459 (cancer-free)	56 Q1: 55 Q2: 56 Q3: 57 Q4: 57	39.4 Q1: 41.9 Q2: 39.1 Q3: 37.9 Q4: 38.5	28,693 cancer deaths	(9)
4	Ke J, 2024	UK	374,792 (cancer-free)	56.2 (8.1) Q1: 54.0 (8.3) Q2: 56.4 (8.1) Q3: 57.2 (7.9) Q4: 57.2 (7.8)	46.5 Q1: 33.0 Q2: 41.7 Q3: 50.1 Q4: 61.0	13,054 cancer deaths	(19)
5	Jochems SHJ, 2023	Sweden	68,147 (cancer-free)	47.3 (10.3)	100	473 prostate cancer deaths	(29)
6	Sun M, 2022	US	9,254 (cancer-free)	Q1: 60.53 (11.39) Q2: 62.38 (11.54) Q3: 62.87 (11.13) Q4: 62.12 (10.66)	Q1: 44.7 Q2: 49.5 Q3: 49.6 Q4: 54.3	352 cancer deaths	(24)
II. The association between TyG index and cancer mortality in cancer-diagnosed people (2)							
1	Fritz J, 2024	Europe	11,760 (prostate cancer)	68.0 (8.3) (age at cancer diagnosis) Q1: 68.1 (8.5) Q2: 68.1 (8.4) Q3: 68.3 (8.3) Q4: 67.8 (8.1)	100	1,784 prostate cancer deaths	(22)
2	Jochems SHJ, 2023	Sweden	3,940 (prostate cancer)	Non-aggressive cancer: 67.2 (6.7) Aggressive cancer: 70.9 (7.3) (age at cancer diagnosis)	100	473 prostate cancer deaths	(29)
III. The association between TyG index and all-cause mortality in cancer-diagnosed people (8)							
1	Yao ZY, 2025	China	300 (advanced gastric cancer treated with sintilimab combined with chemotherapy)	18-75	49.33 Low TyG: 48.78 High TyG: 50.00	–	(32)
2	Li F, 2025	China	335 (breast cancer treated with neoadjuvant chemotherapy)	46	0	–	(35)
3	Liu GM, 2024	China	415 (hepatocellular carcinoma with hepatectomy as initial treatment)	58.0 (11.07) Low TyG: 57.5 High TyG: 60	89.6 Low TyG: 89.5 High TyG: 89.7	51 deaths	(34)
4	Zha B, 2024	US	187 (gastrointestinal cancer)	73.0	49.7	–	(23)
5	Önder T, 2024	Turkey	333 (HR+/HER2- metastatic breast cancer treated with CDK4/6i plus endocrine therapy)	56 Low TyG: 55.3 (12.3) High TyG: 57.9 (11.7)	0	–	(16)
6	Qin G, 2024	China	651 (postoperative renal cell carcinoma cancer)	56	63.3	106 deaths	(25)
7	Cai C, 2024	China	822 (gastric cancer treated with radical resection gastrectomy)	64.65 (10.81) Low TyG: 64.64 (11.48) High TyG: 64.68 (9.88)	73.2 Low TyG: 75.7 High TyG: 70.0	–	(31)
8	Liu XY, 2023	China	571 (female reproductive system cancer)	52 (14)	0	–	(28)

SD, standard deviation; FU, follow-up; TyG, triglyceride-glucose index; CDK 4/6i, cyclin-dependent kinase 4/6 inhibitor.

**Table 2:** Lists of estimates with 95% confidence intervals of the Triglyceride‐glucose index and cancer incidence and mortality.

Author, year	Outcomes	Exposure category	Case/Population	Estimates	Effect metrics
The association between TyG index and cancer incidence (N = 9)					
Wu Z, 2025	Pancreatic cancer	Q1 (≤ 6.72) Q2 (6.72 ~ 7.09) Q3 (7.09 ~ 7.49) Q4 (≥ 7.49) Per SD (0.57) increase		Ref 1.161 (0.999-1.349) 1.234 (1.066-1.429) 1.249 (1.078-1.447) 1.083 (1.031-1.137)	HR
Yang C, 2024	Esophageal cancer	Q1 (≤ 8.5) Q2 (8.5 ~ 8.7) Q3 (8.7 ~ 8.9) Q4 (≥ 8.9) Per SD increase		Ref 0.97 (0.77-1.22) 1.04 (0.84-1.29) 1.13 (0.91-1.40) 1.07 (1.00-1.15)	HR
	Esophageal adenocarcinoma	Q1 (≤ 8.5) Q2 (8.5 ~ 8.7) Q3 (8.7 ~ 8.9) Q4 (≥ 8.9) Per SD increase		Ref 1.21 (0.88-1.65) 1.40 (1.05-1.89) 1.54 (1.15-2.06) 1.16 (1.07-1.26)	
	Esophageal squamous cell carcinoma	Q1 (≤ 8.5) Q2 (8.5 ~ 8.7) Q3 (8.7 ~ 8.9) Q4 (≥ 8.9) Per SD increase		Ref 0.67 (0.46-0.97) 0.57 (0.39-0.85) 0.55 (0.36-0.82) 0.80 (0.67-0.95)	
Son M, 2024	Colorectal cancer	Q1 Q2 Q3 Q4	1266/78,613 1468/77,345 1606/78,955 1772/79,228	Ref 1.08 (1.00-1.16) 1.10 (1.02-1.19) 1.16 (1.07-1.25)	HR
	Colon cancer	Q1 Q2 Q3 Q4		Ref 1.05 (0.95-1.16) 1.09 (0.99-1.20) 1.12 (1.01-1.24)	
	Rectal cancer	Q1 Q2 Q3 Q4		Ref 1.08 (0.93-1.26) 1.22 (1.05-1.41) 1.32 (1.14-1.54)	
Kityo A, 2024	Colorectal cancer	Per 1 increase	699/98,800	1.28 (1.12-1.46)	HR
	Colon cancer	Per 1 increase	422/98,800	1.29 (1.10-1.54)	
	Rectal cancer	Per 1 increase	277/98,800	1.24 (1.01-1.52)	
Jochems SHJ, 2023	Prostate cancer	T1 T2 T3 Per SD (0.5) increase	1165/18,927 1114/18,984 1046/18,986 3325/56,897	Ref 0.96 (0.88-1.04) 0.92 (0.84-1.00) 0.96 (0.88-1.00)	HR
Liu T, 2022	Colorectal cancer	Q1 (≤ 8.19) Q2 (8.19 ~ 8.58) Q3 (8.58 ~ 9.06) Q4 (≥ 9.06) Per 1 increase		Ref 1.13 (0.88-1.45) 1.36 (1.06-1.76) 1.50 (1.19-1.91) 1.19 (1.05-1.34)	HR
Wang L, 2021	Lung cancer	Low TyG (< 8.639) High TyG (≥ 8.639) Per SD (0.541) increase		Ref 0.966 (0.850-1.097) 0.911 (0.640-1.182)	HR
Okamura T, 2020	Colorectal cancer	Per 1 increase	116/27,944	1.38 (1.00-1.91)	HR
Fritz J, 2020	Esophageal adenocarcinoma	Q1 (< 8.1) Q2 (8.1 ~ 8.4) Q3 (8.4 ~ 8.7) Q4 (8.7 ~ 9.1) Q5 (> 9.1) Per SD (0.60) increase	185/510,471	Ref 0.97 (0.58-1.62) 1.09 (0.66-1.80) 1.25 (0.77-2.04) 1.27 (0.77-2.07) 1.11 (0.95-1.29)	HR
	Colon cancer	Q1 (< 8.1) Q2 (8.1 ~ 8.4) Q3 (8.4 ~ 8.7) Q4 (8.7 ~ 9.1) Q5 (> 9.1) Per SD (0.60) increase	4032/510,471	Ref 0.98 (0.88-1.10) 1.07 (0.96-1.19) 1.16 (1.04-1.29) 1.14 (1.03-1.27) 1.07 (1.03-1.10)	
	Rectal cancer	Q1 (< 8.1) Q2 (8.1 ~ 8.4) Q3 (8.4 ~ 8.7) Q4 (8.7 ~ 9.1) Q5 (> 9.1) Per SD (0.60) increase	2430/510,471	Ref 1.04 (0.90-1.19) 1.12 (0.98-1.28) 1.13 (0.99-1.30) 1.24 (1.08-1.42) 1.09 (1.04-1.14)	
	Liver cancer	Q1 (< 8.1) Q2 (8.1 ~ 8.4) Q3 (8.4 ~ 8.7) Q4 (8.7 ~ 9.1) Q5 (> 9.1) Per SD (0.60) increase	561/510,471	Ref 1.13 (0.83-1.54) 1.11 (0.82-1.50) 1.01 (0.74-1.37) 1.29 (0.96-1.72) 1.13 (1.04-1.23)	
	Gallbladder cancer	Q1 (< 8.1) Q2 (8.1 ~ 8.4) Q3 (8.4 ~ 8.7) Q4 (8.7 ~ 9.1) Q5 (> 9.1) Per SD (0.60) increase	364/510,471	Ref 1.23 (0.84-1.81) 1.21 (0.83-1.77) 1.15 (0.79-1.68) 1.38 (0.95-1.99) 1.11 (0.99-1.24)	
	Pancreatic cancer	Q1 (< 8.1) Q2 (8.1 ~ 8.4) Q3 (8.4 ~ 8.7) Q4 (8.7 ~ 9.1) Q5 (> 9.1) Per SD (0.60) increase	1368/510,471	Ref 1.19 (0.98-1.44) 1.20 (1.00-1.46) 1.27 (1.05-1.53) 1.37 (1.13-1.65) 1.12 (1.06-1.19)	
	Pancreatic cancer (male)	Q1 (< 8.1) Q2 (8.1 ~ 8.4) Q3 (8.4 ~ 8.7) Q4 (8.7 ~ 9.1) Q5 (> 9.1) Per SD (0.60) increase	776/510,471	Ref 1.12 (0.88-1.43) 1.19 (0.94-1.52) 1.11 (0.87-1.42) 1.25 (0.98-1.59) 1.08 (1.00-1.16)	
	Pancreatic cancer (female)	Q1 (< 8.1) Q2 (8.1 ~ 8.4) Q3 (8.4 ~ 8.7) Q4 (8.7 ~ 9.1) Q5 (> 9.1) Per SD (0.60) increase	592/510,471	Ref 1.32 (0.96-1.81) 1.24 (0.90-1.70) 1.54 (1.13-2.08) 1.58 (1.16-2.14) 1.19 (1.09-1.31)	
	Breast cancer (postmenopausal)	Q1 (< 8.1) Q2 (8.1 ~ 8.4) Q3 (8.4 ~ 8.7) Q4 (8.7 ~ 9.1) Q5 (> 9.1) Per SD (0.60) increase	3427/510,471	Ref 1.04 (0.92-1.18) 1.08 (0.96-1.22) 1.07 (0.95-1.20) 1.07 (0.95-1.20) 1.02 (0.98-1.07)	
	Endometrium cancer	Q1 (< 8.1) Q2 (8.1 ~ 8.4) Q3 (8.4 ~ 8.7) Q4 (8.7 ~ 9.1) Q5 (> 9.1) Per SD (0.60) increase	1417/510,471	Ref 1.27 (1.05-1.54) 1.07 (0.88-1.29) 1.28 (1.06-1.54) 1.22 (1.01-1.47) 1.04 (0.98-1.11)	
	Ovary cancer	Q1 (< 8.1) Q2 (8.1 ~ 8.4) Q3 (8.4 ~ 8.7) Q4 (8.7 ~ 9.1) Q5 (> 9.1) Per SD (0.60) increase	921/510,471	Ref 1.03 (0.83-1.27) 0.89 (0.72-1.11) 1.04 (0.84-1.29) 1.00 (0.80-1.25) 1.00 (0.92-1.08)	
	Kidney renal cell cancer	Q1 (< 8.1) Q2 (8.1 ~ 8.4) Q3 (8.4 ~ 8.7) Q4 (8.7 ~ 9.1) Q5 (> 9.1) Per SD (0.60) increase	1347/510,471	Ref 1.06 (0.87-1.28) 1.02 (0.84-1.23) 1.18 (0.98-1.42) 1.36 (1.13-1.63) 1.13 (1.07-1.20)	
	Digestive organs cancer Combined	Q1 (< 8.1) Q2 (8.1 ~ 8.4) Q3 (8.4 ~ 8.7) Q4 (8.7 ~ 9.1) Q5 (> 9.1) Per SD (0.60) increase	8940/510,471	Ref 1.04 (0.97-1.12) 1.11 (1.03-1.19) 1.16 (1.08-1.24) 1.22 (1.14-1.31) 1.09 (1.06-1.11)	
	Endometrium, ovary and breast (postmenopausal) cancer Combined	Q1 (< 8.1) Q2 (8.1 ~ 8.4) Q3 (8.4 ~ 8.7) Q4 (8.7 ~ 9.1) Q5 (> 9.1) Per SD (0.60) increase	5765/510,471	Ref 1.09 (1.00-1.20) 1.05 (0.96-1.15) 1.11 (1.02-1.22) 1.09 (1.00-1.20) 1.03 (0.99-1.06)	
The association between TyG index and cancer mortality in cancer-free people (6)					
Li S, 2024	Cancer death	Per SD (0.69) increase Per 1 increase		1.18 (1.03-1.36) 1.28 (1.05-1.56)	HR
Fritz J, 2024	Prostate cancer death	Q1 Q2 Q3 Q4 Per SD (0.6) increase	1784/259,884	Ref 0.99 (0.86-1.13) 1.05 (0.92-1.21) 1.00 (0.87-1.15) 1.03 (0.94-1.13)	HR
He G, 2024	Cancer death	Q1 (< 8.41) Q2 (8.41 ~ 8.76) Q3 (8.76 ~ 9.16) Q4 (≥ 9.16) Per 1 increase	7634/880,296 7399/883,137 6944/879,621 6716/881,405 28693/3,524,459	Ref 0.99 (0.96-1.03) 0.97 (0.94-1.01) 0.94 (0.91-0.98) 0.97 (0.94-0.99)	HR
Ke J, 2024	Cancer death	Q1 (< 8.31) Q2 (8.31 ~ 8.67) Q3 (8.68 ~ 9.07) Q4 (≥ 9.08)	2998/93,478 2448/92,728 3616/94,421 3992/94,165	1.06 (1.01-1.12) Ref 1.05 (1.00-1.11) 1.07 (1.02-1.13)	HR
Jochems SHJ, 2023	Prostate cancer death	T1 T2 T3 Per SD increase	121/18,927 139/18,984 147/18,986 407/56,897	Ref 1.13 (0.89-1.45) 1.24 (0.97-1.62) 1.14 (0.92-1.39)	HR
Sun M, 2022	Cancer death	Q1 (≤ 8.32) Q2 (8.32 ~ 8.72) Q3 (8.72 ~ 9.16) Q4 (> 9.16) Per 1 increase	71/2,306 95/2,330 88/2,318 98/2,300 352/9,254	Ref 1.08 (0.78-1.49) 1.05 (0.75-1.46) 0.94 (0.66-1.34) 0.92 (0.76-1.11)	HR
The association between TyG index and cancer mortality in cancer-diagnosed people (2)					
Fritz J, 2024	Prostate cancer death	Q1 Q2 Q3 Q4 Per SD (0.6) increase	1784/11,760	Ref 1.03 (0.90-1.18) 1.15 (1.00-1.32) 1.17 (1.01-1.35) 1.11 (1.01–1.22)	HR
Jochems SHJ, 2023	Prostate cancer death	T1 T2 T3 Per SD increase	102/1,022 125/1,042 123/1,028 350/3,092	Ref 1.25 (0.95-1.65) 1.38 (1.04-1.82) 1.24 (1.00-1.55)	HR
The association between TyG index and all-cause mortality in cancer-diagnosed people (8)					
Yao ZY, 2025	Advanced gastric cancer treated with sintilimab combined with chemotherapy death	Low TyG (< 1.79) High TyG (≥ 1.79)	NA	Ref 0.36 (0.24-0.55)	HR
Li F, 2025	Breast cancer treated with neoadjuvant chemotherapy death	Low TyG (< 8.01) High TyG (≥ 8.01)	NA	Ref 3.206 (1.328-7.737)	HR
Liu GM, 2024	Hepatocellular carcinoma with hepatectomy as initial treatment death	Low TyG (< 6.58) High TyG (≥ 6.58)	NA	2.43 (1.39-4.24) Ref	HR
Zha B, 2024	Gastrointestinal cancer death	Q1 Q2 Q3 Q4	NA	Ref 1.664 (0.785-3.526) 1.413 (0.649-3.079) 1.296 (0.631-2.661)	HR
Önder T, 2024	HR+/HER2- metastatic breast cancer treated with CDK4/6i plus endocrine therapy death	Low TyG (< 8.43) High TyG (≥ 8.43)	NA	0.513 (0.281-0.936) Ref	HR
Qin G, 2024	Postoperative renal cell carcinoma death	Per SD (4.31) increase	NA	1.729 (1.150-2.60)	HR
Cai C, 2023	Gastric cancer death	Low TyG (≤ 1.4) High TyG (> 1.4)	NA	Ref 0.70 (0.54-0.89)	HR
Liu XY, 2023	Female reproductive system cancer death	Low TyG (≤ 4.62) High TyG (> 4.62) Per 1 increase	NA	Ref 1.49 (1.02-2.17) 1.11 (0.95-1.30)	HR

SD, standard deviation TyG, triglyceride-glucose index Ref, reference HR, hazard ratio OS, overall survival NA, not applied.

### 2.4 Quality assessment

The Newcastle-Ottawa Scale (NOS) was employed for the quality evaluation of the included studies (10). The score assigned to each study ranges from 1 to 9, determined by the quality of cohort selection, the comparability between groups, and the assessment of outcomes. Studies with a total NOS score of 7 or higher were defined as high quality. Any disagreements were resolved by the third reviewer. The details of the NOS assessment of individual studies are shown in **Supplementary Table 4**.

### 2.5 Statistical analysis

Statistical analyses were conducted with R [version 4.4.3]. We utilized the adjusted hazard ratios (HRs) and their corresponding 95% confidence intervals (CIs) as the general indicator of the correlation between the TyG index and cancer-associated outcomes. The TyG index is calculated as Ln(Fasting Triglycerides [mg/dL] × [Fasting Plasma Glucose [mg/dL]/2) (5). For studies analyzing the TyG index as a categorial variable, the HRs in the highest group and the lowest group were calculated for comparison. In studies analyzing the TyG index as a continuous variable, the HRs reflecting the risk per standard deviations (SD) or per unit increasement of the TyG index were calculated.

Regardless of the heterogeneity level of the studies, we used random-effect meta-analyses (restricted maximum likelihood [REML]) to collect the HRs for cancer incidence or cancer-related mortality obtained by individual studies for comparison between the highest TyG group and the lowest group, in addition to the HRs for per SD or per unit increasement in the TyG index. And the evaluation of heterogeneity between studies was conducted through Cochrane’s Q test and the I^2^ statistic. I^2^>50% or P<0.1 demonstrated the existence of significant heterogeneity (11). We adopted the random-effects model to merge the potential heterogeneity among the included studies (12). P<0.05 was identified as statistically significant during the analyses. Subgroup analyses were conducted to investigate the possible origins of heterogenicity and identify the specific association between the TyG index and some typical outcomes. Additionally, the evaluation of the publication bias was conducted using funnel plots and Egger’s test (13, 14). Moreover, to verify the stability and reliability of our results, the sensitivity analyses were also performed (15).

## 3 Results

### 3.1 Literature review

**Figure 1:**
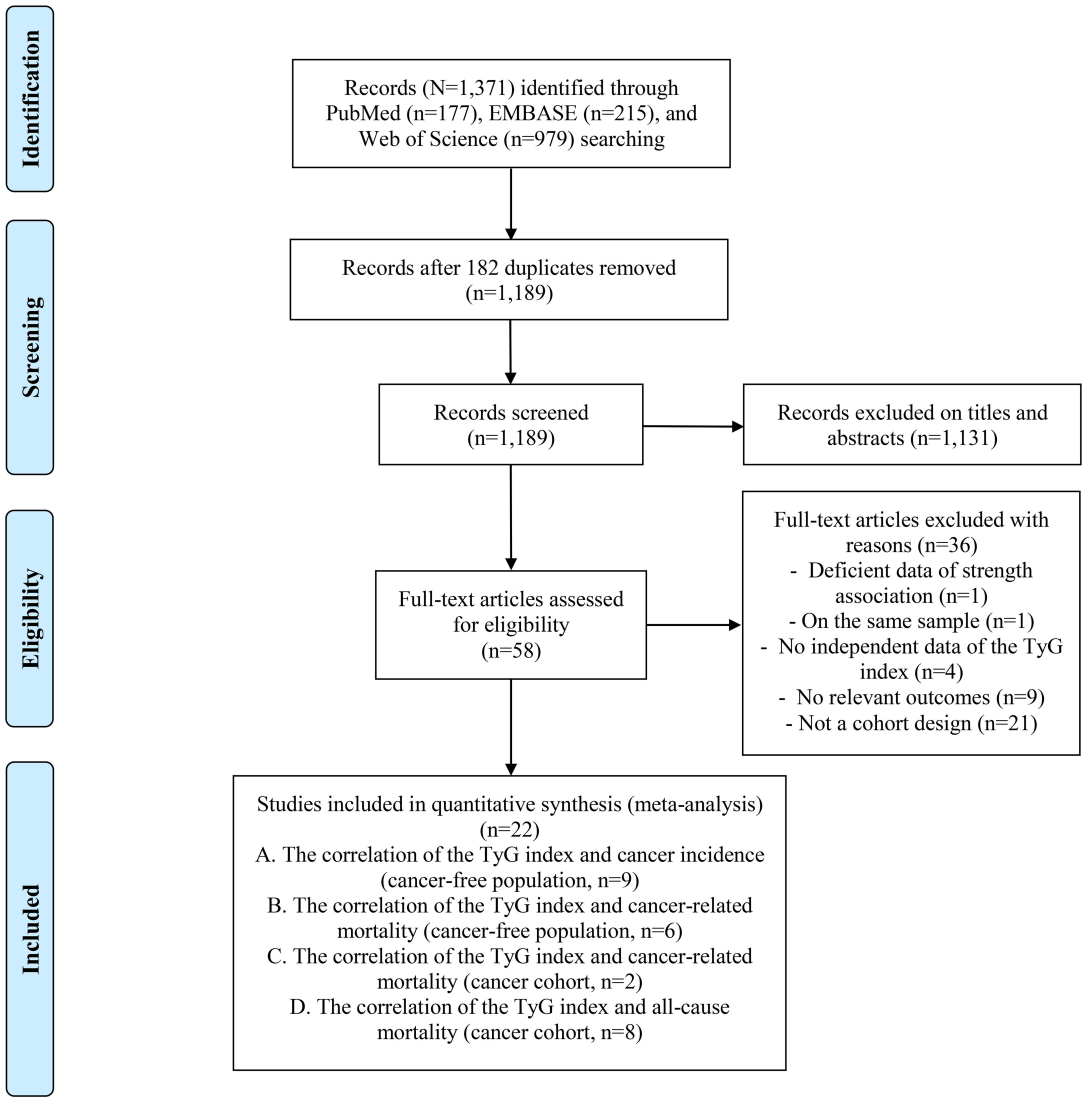
Flowchart of the database search and study screening.

A total of 1,371 records were obtained from the initial search in three databases. Among these, 182 were removed due to duplication. Subsequently, 1,131 records were excluded during the title and abstract screening step, and 36 were removed during the full-text assessment for specific reasons. All excluded studies for reasons (n=36) are shown in **Supplementary Table 3**. Ultimately, there were 22 included articles meeting all the inclusion criteria for this meta-analysis (**Figure 1**) (7, 9, 16–35), which estimated the correlation between the TyG index and cancer-associated outcomes, respectively cancer incidence (n=9) (7, 17, 18, 20, 21, 27, 29, 30, 33), cancer-related mortality in cancer-free people (n=6) (9, 19, 22, 24, 26, 29), cancer-related mortality in cancer-diagnosed people (n=2) (22, 29), and all-cause mortality in cancer patients (n=8) (16, 23, 25, 28, 31, 32, 34, 35).

### 3.2 Study characteristics

**Table 1** summarizes the basic characteristics of the included studies, including the first author, publication year, country, sample size, follow-up (FU) period, baseline age and sex ratio of participants, the TyG index analysis pattern, reported outcomes, and the assessment of outcomes. These studies were conducted in China (9, 17, 25, 28, 31, 32, 34, 35), the UK (18, 19, 30, 33), Korea (20, 21), Sweden (29), Japan (27), Turkey (16), and the US (23, 24, 26). The sample sizes of included studies varied from 187 to 3,524,459, although the outcomes differed. The mean age of the participants in individual studies ranged from 43.1 to 73, with the male ratio varying from 33.45 to 89.6%, except for some studies focusing on male/female specialized cancers (16, 22, 28, 29, 35). The median FU duration of included studies ranged from 2.39 to 17.5 years. Due to distinctively different cohort and outcome selection, studies included for the analysis of correlation between the TyG index and all-cause mortality in cancer patients have relatively smaller sample sizes, older mean age of participants, and shorter follow-up duration. Almost all the cancer diagnoses in these studies were validated by the International Classification of Diseases (ICD), except for one study using gastroenterologist clinical diagnosis as the outcome validation (27). **Table 2** mainly summarizes the estimates with 95% CI of the TyG index and different outcomes in individual studies, along with descriptions of adjustments made in individual studies.

### 3.3 Quality assessment

All studies included in this meta-analysis were cohort studies. Their quality was evaluated using the NOS scale with a maximum score of 9 points. For included studies with cancer-free people participation (n=14), only one study with a score of 5 was unqualified to be assessed as high quality (≥7). And for included studies with cancer-diagnosed people participation (n=10), only one study with a score of 6 was insufficient to be categorized as high quality.

### 3.4 Association between the TyG index and the risk of cancer incidence

**Figure 2:**
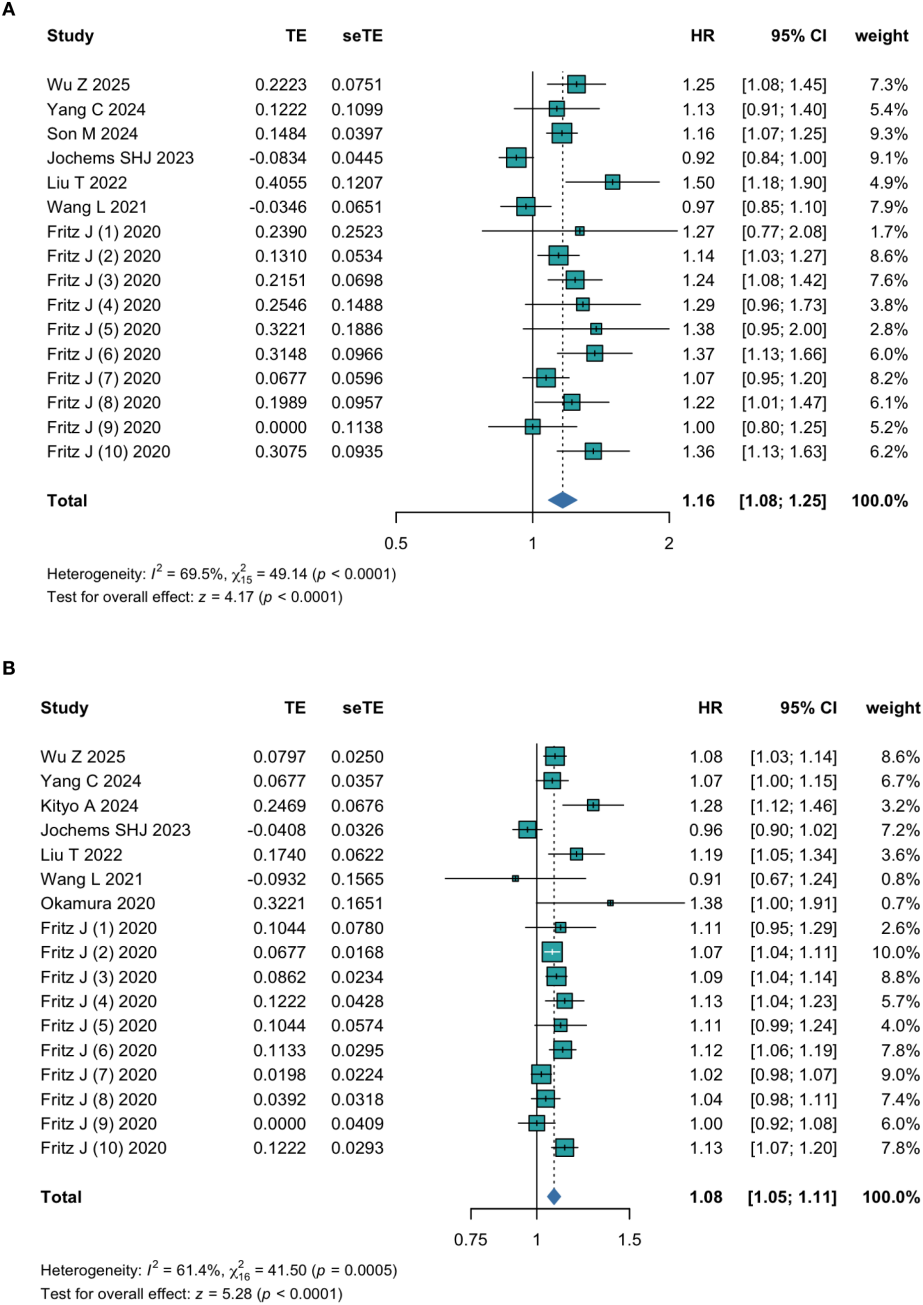
The pooled result of meta-analysis of cancer risk (**(A)** Categorized; **(B)** Continuous).

There were seven studies analyzing the TyG index as a categorial variable, and the collective results from these studies showed a significant increase in the cancer occurrence during the follow-up period among the participants in the highest TyG index group compared with those in the lowest TyG index group (HR = 1.16; 95% CI 1.08-1.25; I^2^ = 69.5%, P<0.01 **Figure 2A**). This finding aligned with the results of the meta-analysis in which the TyG index was analyzed as a continuous variable (HR = 1.08; 95% CI 1.05-1.11; I^2^ = 61.4%, P<0.01 **Figure 2B**). Applying the leave-one-out sensitivity analysis did not significantly change the pooled results from incidence cohorts, indicating that no single study exserted a disproportionate influence and the results are reliable (**Supplementary Figure 2**).

Additionally, the subgroup analysis mainly analyzed the relationship between the TyG index and the incidence of several specific obesity-related cancers with more extensive discussion in the literature. The results identified a significant clinical association between the TyG index and some obesity-related cancer incidence, which including colon cancer (HR = 1.13; 95% CI 1.05-1.22; P>0.05 **Supplementary Figure 1**), rectal cancer (HR = 1.28; 95% CI 1.15-1.41; P>0.05 **Supplementary Figure 1**) and pancreatic cancer (HR = 1.29; 95% CI 1.15-1.45; P>0.05 **Supplementary Figure 1**). However, no such association was observed for esophageal cancer (HR = 1.15; 95% CI 0.94-1.40; P>0.05 **Supplementary Figure 1**). Based on the consensus that there is a close association between the TyG index and IR, current studies on the relationship between the TyG index and cancer have primarily focused on specific obesity-related cancers, such as colorectal and pancreatic cancers. Consequently, more clinical studies are available for these cancer types, allowing for further aggregation and analysis. However, other specific cancer types have not been extensively studied, or the results concerning these outcomes have been insufficient for robust subsequent analyses.

### 3.5 Association between the TyG index and the risk of cancer-related mortality among cancer-free people and cancer-diagnosed people

**Figure 3:**
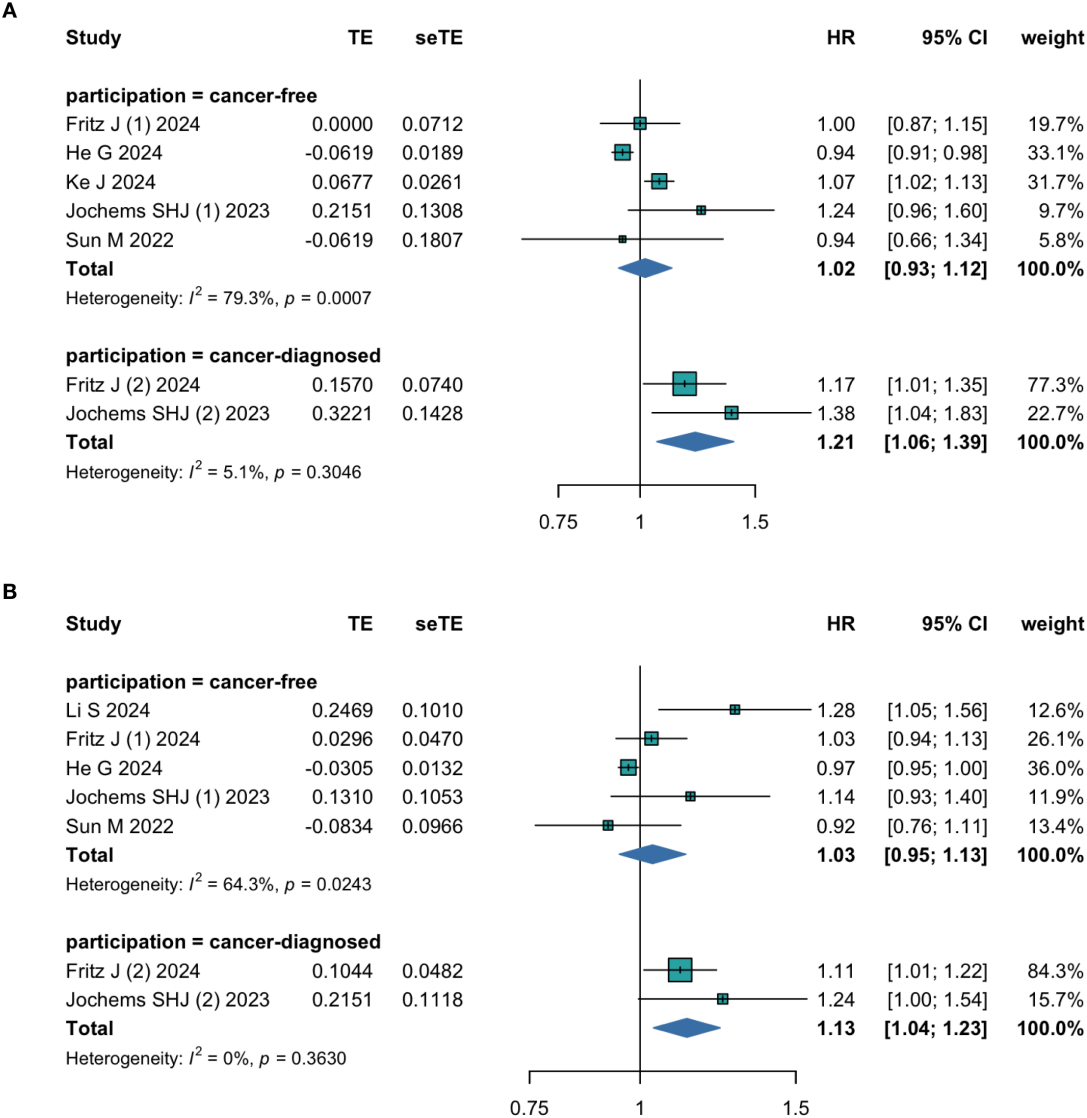
The pooled result of meta-analysis of cancer-related mortality among cancer-free and cancer-diagnosed people (**(A)** Categorized; **(B)** Continuous).

The meta-analysis of five studies with the participation of cancer-free people showed that there was no significant association between the TyG index and cancer-related mortality when treated the TyG index as a categorial variable (HR = 1.02; 95% CI 0.93-1.12; I^2^ = 79.3%, P<0.01 **Figure 3A**) or as a continuous variable (HR = 1.03; 95% CI 0.95-1.13; I^2^ = 64.3%, P<0.05 **Figure 3B**). The sensitivity analyses by excluding one study at a time showed consistent results (95% CI for the TyG index analyzed as a categorial variable: 0.93–1.12, P > 0.05; 95% CI for the TyG index analyzed as a continuous variable: 0.95-1.13, P>0.05 **Supplementary Figure 3**). However, the sensitivity analysis of studies examined the TyG index as a categorial variable also identified that, once omitting the study conducted by He G et al. (9), a significant association between the TyG index and relevant outcome among rest studies was observed, and the heterogeneity between studies also disappeared (HR = 1.06; 95% CI 1.02-1.12; P<0.01 **Supplementary Figure 3**). Since such association not emerged in studies analyzed the TyG index as a continuous variable (HR = 1.07; 95% CI 0.95-1.21; P>0.05 **Supplementary Figure 3**), the conclusion concerning the association between the TyG index and cancer-related mortality among cancer-free people should be drawn with caution. The details of the forest plots after removing this study are shown in **Supplementary Figure 8** (estimates for the TyG index analyzed as a categorical variable: HR = 1.06, 95% CI 1.02–1.12, I^2^ = 0%, P > 0.05; estimates for the TyG index analyzed as a continuous variable: HR = 1.07, 95% CI 0.95-1.21, I^2^ = 53.7%, P<0.05 **Supplementary Figure 8**).

Furthermore, we conducted the subgroup analysis based on the specific death reason among cancer-free people. Alos, the results identified that there was no significant association between the TyG index and cancer-related mortality among cancer-free people neither in the subgroup with the general cancer death as the outcome (estimates for the TyG index analyzed as a categorical variable: HR = 1.00, 95% CI 0.89–1.12, I^2^ = 87.6%, P < 0.01; estimates for the TyG index analyzed as a continuous variable: HR = 1.03, 95% CI 0.88-1.21, I^2^ = 74.2%, P<0.05 **Supplementary Figure 9**) nor in the subgroup with the prostate cancer death as the outcome (estimates for the TyG index analyzed as a categorical variable: HR = 1.08, 95% CI 0.88–1.33, I^2^ = 52.1%, P > 0.05; estimates for the TyG index analyzed as a continuous variable: HR = 1.05, 95% CI 0.96-1.14, I^2^ = 0%, P>0.05 **Supplementary Figure 9**), which were coherent with the primitive meta-analysis and partially explained the heterogenicity detected.

Besides, the pooled result of two cohort studies showed that a higher TyG index was significantly associated with a higher risk of cancer-related mortality among cancer-diagnosed people. This association was significant whether the TyG index was treated as a categorial variable (HR = 1.21; 95% CI 1.06-1.39; P>0.05 **Figure 3A**) or as a continuous variable (HR = 1.13; 95% CI 1.04-1.23; P>0.05 **Figure 3B**). Given the limited number of studies in the literature, the pooled results should be interpreted with caution and more studies in this field are needed to substantiate the association between the TyG index and cancer-related mortality among cancer-diagnosed people.

### 3.6 Association between the TyG index and the risk of all-cause mortality in cancer patients

**Figure 4:**
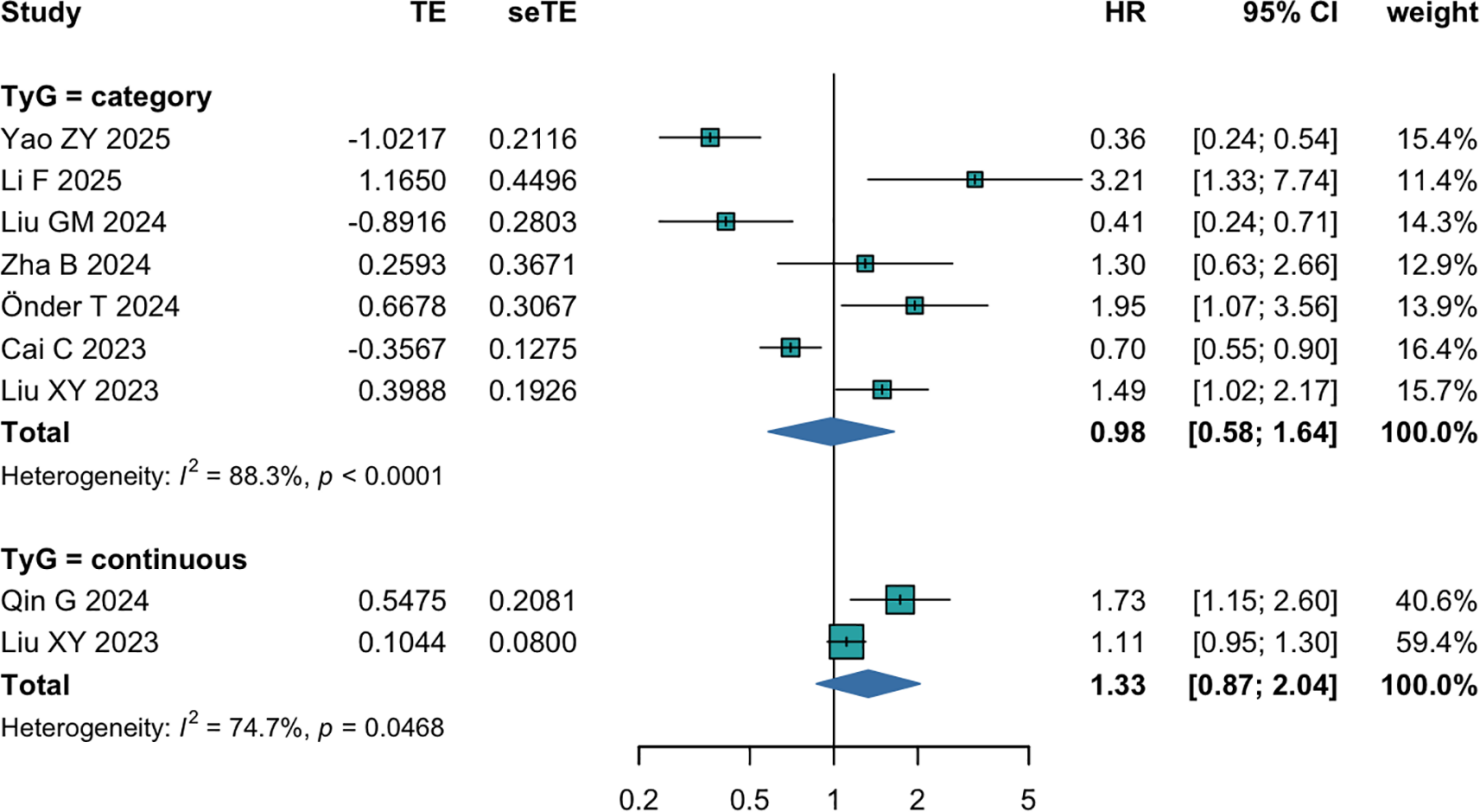
The pooled result of the meta-analysis of all-cause mortality among cancer patients.

The pooled results of seven studies showed that there was no significant association between the TyG index and all-cause mortality among cancer patients when the TyG index was analyzed as a categorial variable (HR = 0.98; 95% CI 0.58-1.64; I^2^ = 88.3%, P<0.01 **Figure 4**). The sensitivity analysis demonstrated that there were no extreme results influencing the overall risk estimates (HR = 0.98; 95% CI 0.58-1.64; I^2^ = 88.3%, P>0.05 **Supplementary Figure 4**), confirming the robustness and reliability of the results.

When analyzed the TyG index as a continuous variable, with the inclusion of only two studies, the pooled results did not observe a prominent association between the TyG index and all-cause mortality in cancer patients either (HR = 1.33; 95% CI 0.87-2.04; I^2^ = 74.7%, P<0.05 **Figure 4**). Considering the limited available number of studies included, the results should be interpreted cautiously.

### 3.7 Publication bias

Funnel plots for cancer incidence, cancer-related mortality in cancer-free people, and all-cause mortality in cancer patients are shown in **Supplementary Figures 5–7**. Visual inspection of the funnel plots for cancer-related mortality studies and all-cause mortality studies showed symmetry. Although the plot for cancer incidence studies revealed asymmetry, the potential missing studies were broadly in the gray area of significance for the plot. Therefore, it is unlikely that publication bias is the major reason for the observed asymmetry. Egger’s test was also conducted to further evaluate publication bias (**Supplementary Figures 5–7**). Test results suggested no significant publication bias among the included studies for studies assessing cancer incidence (P = 0.0584), cancer-related mortality among cancer-free people (P = 0.5376), and all-cause mortality among cancer patients (P = 0.3782).

## 4 Discussion

In this meta-analysis of cohort studies, people in the highest TyG index category showed a higher risk of cancer incidence than those in the lowest TyG index category, which was similar to previous research (36). Subgroup analyses showed that the association between the TyG index and the occurrence of obesity-related cancers, such as colon cancer and pancreatic cancer, remains significant. Besides, meta-analysis of studies analyzing the TyG index as a continuous variable also showed similar results. In summary, these results suggested that a higher TyG index may be a potential predictor of an increased risk of cancer incidence in the general population.

Besides, this study was the first meta-analysis to summarize the association between the TyG index and cancer prognosis outcomes. Although we had three different cancer prognosis outcomes investigated, the significant association between the TyG index and cancer prognosis was only observed in the meta-analysis of two studies examining the TyG index and cancer-related mortality among cancer-diagnosed people. Several discrepancies have been observed among the studies investigating the association between the TyG index and all-cause mortality in cancer patients. Variations in cancer sites, cohort selection, adjustments for confounding factors, and sample sizes may influence the meta-analysis results concerning the association between the TyG index and specific outcomes accordingly. Thus, more studies in this field are needed to investigate the association between the TyG index and cancer prognosis.

While the exact mechanism by which the TyG index influences cancer risk is not fully understood, several possible mechanisms related to IR have been suggested. First, IR leads to hyperinsulinemia, which increases IGF-1 levels (36). Research has demonstrated that IGF-1 may facilitate tumor development by stimulating cellular proliferation and inhibiting the process of apoptosis (37–39). This IR-driven pathway activates oncogenic signaling, specifically the PI3K/Akt/mTOR pathway, thereby facilitating tumor progression and contributing to resistance against therapeutic interventions (17). Furthermore, an elevated TyG index is correlated with chronic inflammation, which is characterized by heightened levels of pro-inflammatory cytokines, including interleukin-6 (IL-6) and tumor necrosis factor-alpha (TNF-α). These cytokines contribute to DNA damage, cellular proliferation, and inhibition of apoptosis, thereby promoting the process of carcinogenesis (40). Besides, an elevated TyG index is also associated with oxidative stress, which could induce DNA damage and mutations to promote the development of cancers (40). Additionally, as a result of IR, dysregulation of adipokines, including increased chemerin and decreased adiponectin, fosters a tumorigenic microenvironment through altering metabolism and immune responses (40). Furthermore, abnormal blood sugar levels could elevate oxidative stress, promote chronic inflammation, and establish a pro-angiogenic and anti-apoptotic microenvironment. These conditions may contribute to the processes of carcinogenesis and the progression of cancer (41, 42).

Besides, the TyG index may also act as a surrogate indicator for abnormal lipid metabolism, reflecting its link to cancer development and prognosis. Research has shown that tumor cells often increase *de novo* fatty acid synthesis, lipid uptake, and fatty acid oxidation to support rapid growth and survival in nutrient- and oxygen-deprived environments, which is crucial for cancer progression (43–46). Additionally, dysregulated lipid metabolism influences the tumor microenvironment by altering immune and stromal cell functions, promoting immune evasion, and aiding metastasis (43–46). These lipid alterations may also serve as promising targets for developing biomarkers and therapeutic strategies for cancer diagnosis and treatment (46).

As for the cancer prognosis, some other TyG-related indices such as TyG-waist circumstance and TyG-waist-to-height ratio are also correlated with a higher risk of cancer-related mortality, especially among people diagnosed with prediabetes and gastrointestinal cancers (47, 48). Also, the C-reactive protein-TyG index, serving as a composite biomarker of systemic inflammation and metabolic dysfunction, had been shown to efficiently improves the prognostic prediction for gastrointestinal cancer survival (48).

### 4.1 Strengths and limitations

This meta-analysis had several strengths. First, this meta-analysis included only cohort studies, avoiding potential bias associated with cross-sectional designs. Additionally, only multivariate adjusted estimates were included to reflect the statistical significance of studies with high accuracy. Moreover, meta-analyses were conducted separately with the TyG index analyzed as a categorial variable as well as a continuous variable, and comparable results were achieved, thereby validating the robustness and reliability of the findings. Despite the presence of significant heterogeneity, sensitivity analyses were performed, which yielded consistent and robust results. Importantly, our study is clinically relevant as the TyG index demonstrates strong potential for cancer prevention, diagnosis, and possibly prognosis. Moreover, the novelty of this study lies in that it was the first meta-analysis to summarize the association between the TyG index and cancer prognosis outcomes. Besides the prognosis-related outcomes were specifically discussed comprehensively under three different situations, which was also unprecedented in this field.

Several limitations of this meta-analysis should be considered carefully. First, a limited number of studies in the literature were available for this meta-analysis. And significant heterogeneity emerged across studies on different outcomes, which could be attributed to the differences in cohort selections, cancer sites, and adjustment for covariates. Since different covariates adopted in different studies were not accessible to coordinate for the further subgroup analyses, especially for the analysis of the relationship between the TyG index and all-cause mortality among cancer-diagnosed people, we did not figure out the appropriate method to conduct the further specific analysis or could just combine the results together roughly. In addition, the sample sizes of the included studies varied significantly. Despite the utilization of appropriate meta-analytic methodology with random-effect models, moderate to high levels of heterogeneity persist. Hence, the pooled results for the effect of the TyG index on three cancer-associated outcomes should be interpreted cautiously. Second, the possibility of unidentified articles in other databases remains a limitation of this study. But the comprehensive search of PubMed, Web of Science, and Embase for studies published should have encompassed the majority of all accessible reports. The studies included were mainly from Europe and Asia, which limits their global applicability. Studies in other countries and regions with different ethnicities are especially needed. Fourth, some studies with relatively lower NOS scores were also included, which may influence the final average estimates. However, the reliability and validity of these studies have been thoroughly assessed and found to be acceptable, thereby supporting the accuracy and facticity of the results obtained. Fifth, due to the incomplete statistical information in some studies that analyzed the TyG index as a continuous variable, the HRs reflecting the risk per SD or per unit increasement in the TyG index were pooled directly without any additional adjustments. This approach may have unknown effects on the variation in the results. Sixth, as for the evaluation of the publication bias, both the Egger’s test and the Begg’s test have its inherent limitations. Since the heterogenicity detected among studies in this research was substantial and the amount of included studies was limited, we adopted the Egger’s test supplemented by the funnel plots to examine the publication bias of the included research, although this method may also have limitations to some extent. Seventh, included studies for the analyses of cancer occurrence, cancer-related death among cancer-free people and cancer-diagnosed people have no specific description about the specific treatment for the cohorts, thus we cannot provide more concrete correlated information. Besides, the included studies for the analysis of all-cause mortality among cancer-diagnosed people have substantially significant signatures in both cancer types and corresponding treatments, which were not accessible for the grouping analysis. Eighth, since this field of research remains relatively novel, and existing research still exhibits considerable heterogeneity, with insufficient studies to support practical clinical application, no efficient or significant GRADE evaluation can be conducted to assess the certainty of evidence. Nineth, lack of standardized TyG index cut-off values for cancer occurrence and cancer-related mortality risk stratification, limited prospective data on TyG index utility in diverse population remain research gaps in this study and in this field. Finally, as a shared shortcoming of such meta-analyses, the results of this study could not interpret the causal associations between the TyG index and cancer-associated outcomes. Thus, further basic research is needed to explain the possible mechanisms underlying these associations.

## 5 Conclusions

In summary, our findings suggested that a higher TyG index has adverse effects on cancer incidence but has no confirmable association with cancer prognosis. Given the high prevalence of IR in the general population, especially among cancer patients, screening IR is significant in both clinical and public health contexts, particularly regarding cancer prevention and screening. More studies with a larger scale are needed to investigate the association between the TyG index and cancer prognosis, and further basic studies are needed to investigate the detailed underlying mechanisms that link the TyG index with cancer-associated outcomes.

## Data Availability

The original contributions presented in the study are included in the article/**Supplementary Material**. Further inquiries can be directed to the corresponding author.
