# Supplementary material for: The association of triglyceride-glucose index with cancer incidence and mortality: a systematic review and meta-analysis of cohort studies
Source: Front Endocrinol (Lausanne). 2025 Oct 24;16:1682062. doi: 10.3389/fendo.2025.1682062 (PMC12591978; doi:10.3389/fendo.2025.1682062)

**Supplementary Figures**

**Supplementary Figure 1.** Sub-group analysis of the association between TyG index and the occurrence of some specific obesity-related cancers (A. categorized; B. continuous).

A.
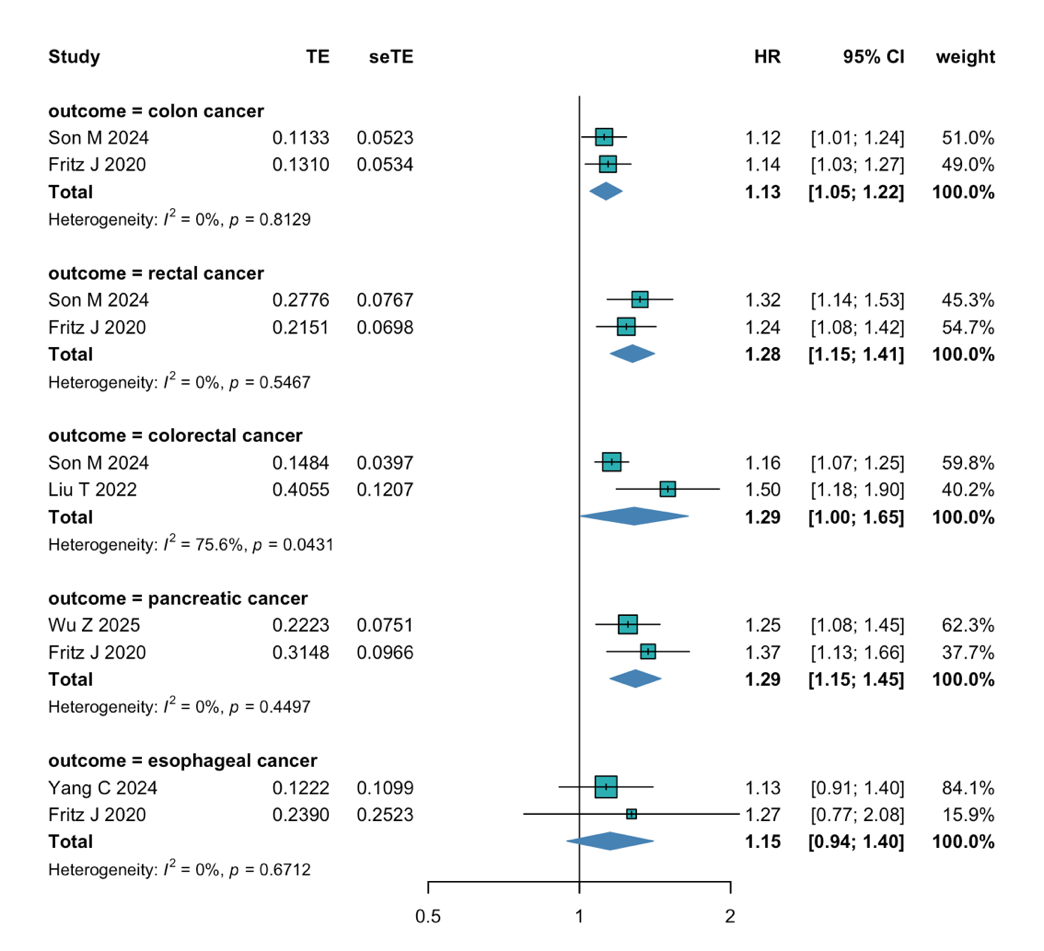


B.
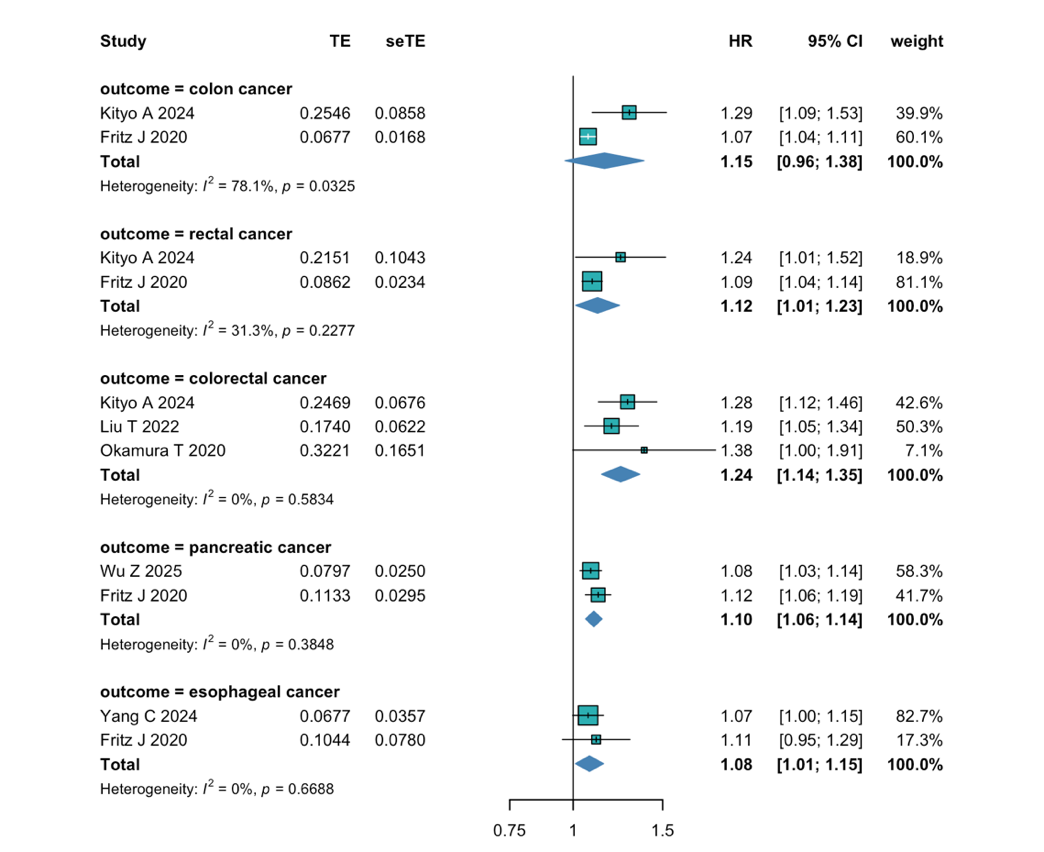


**Supplementary Figure 2.** Sensitivity analysis of the TyG index on the risk of cancer incidence (A. categorized; B. continuous).

A.
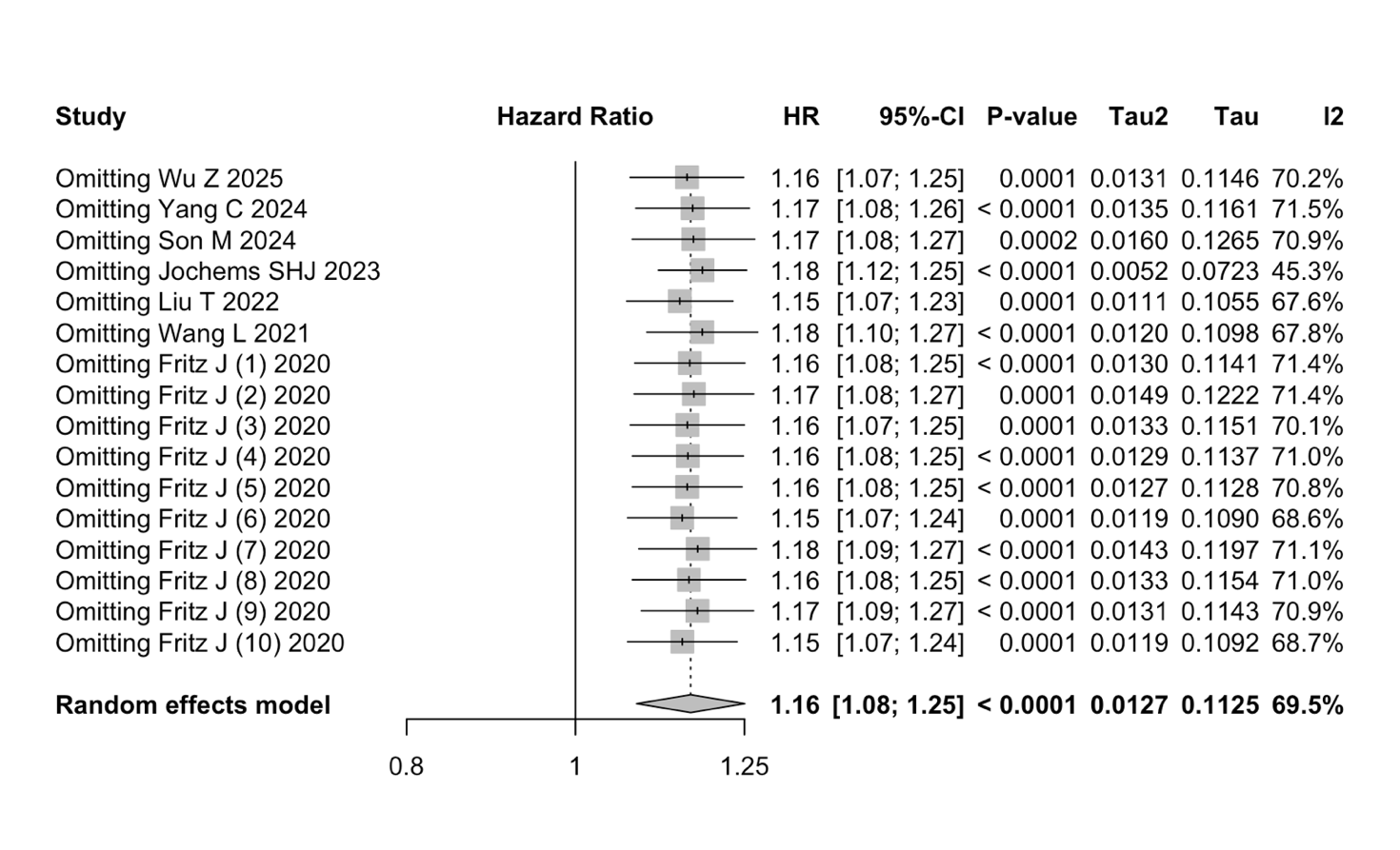


B.
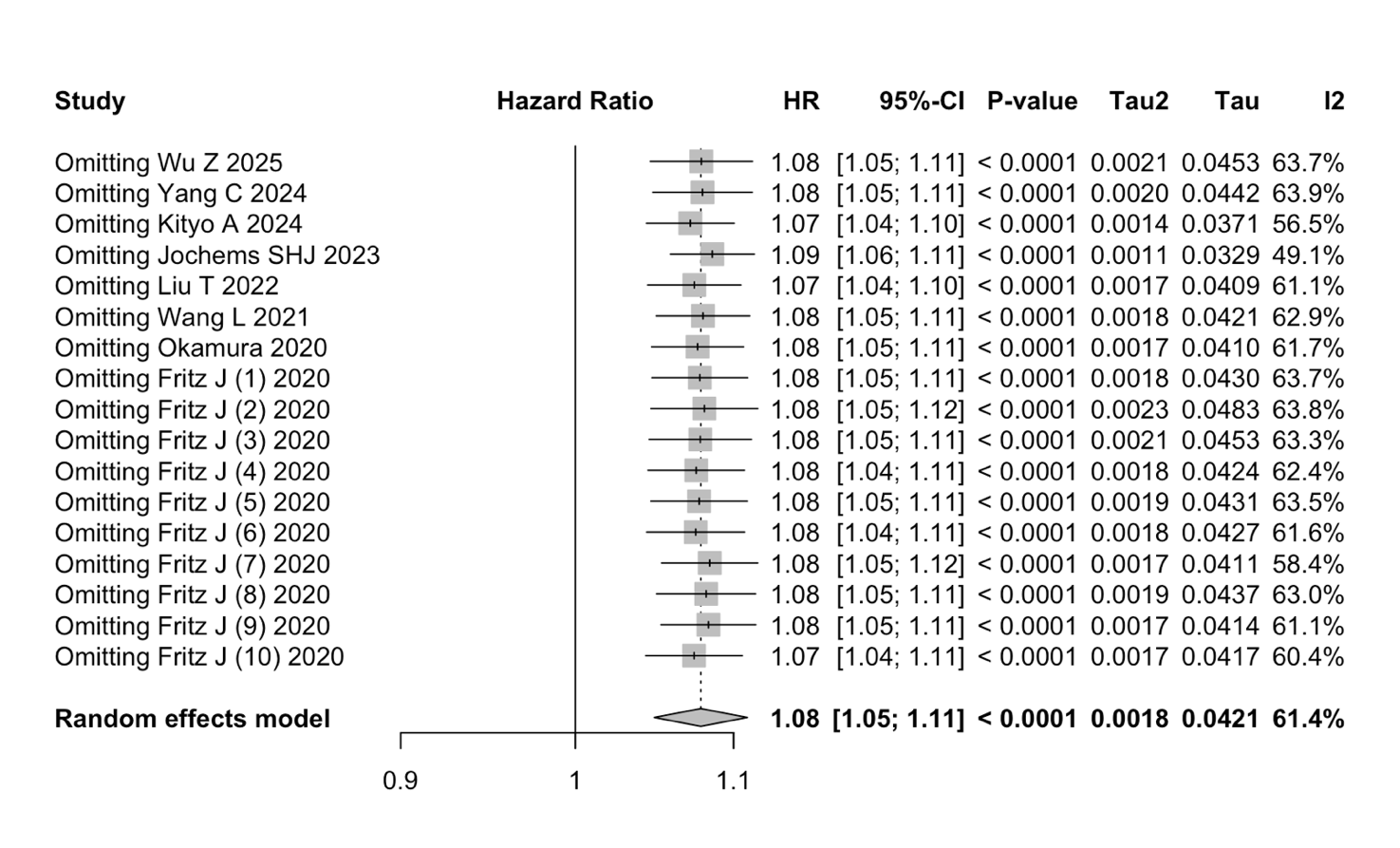


**Supplementary Figure 3.** Sensitivity analysis of the TyG index on the risk of cancer-related mortality among cancer-free people (A. categorized; B. continuous).

A.
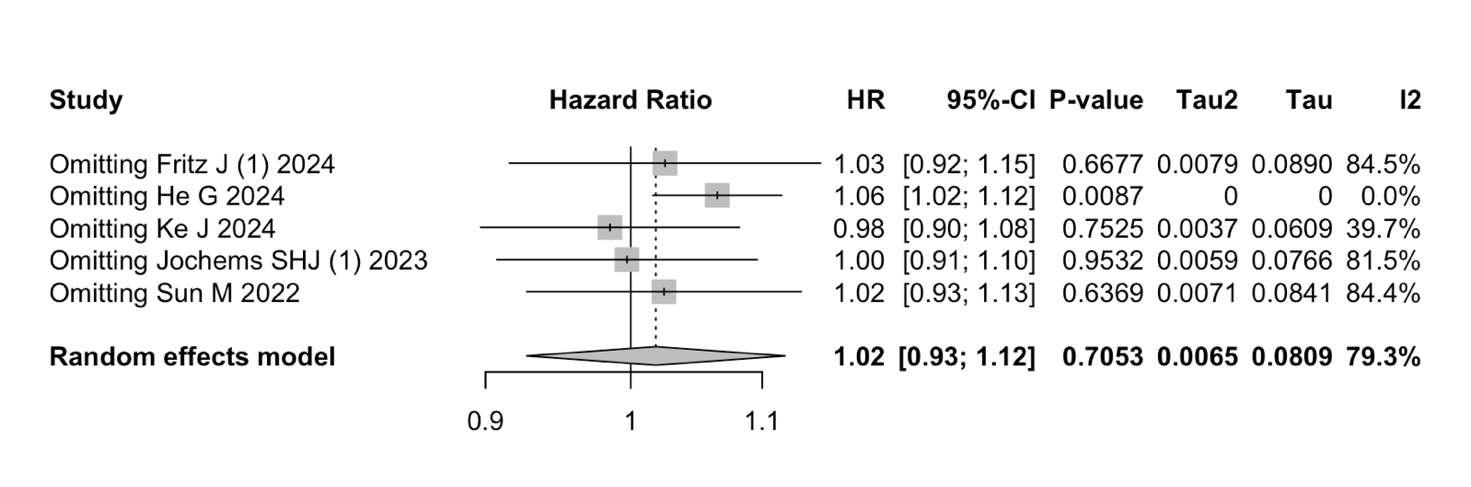


B.
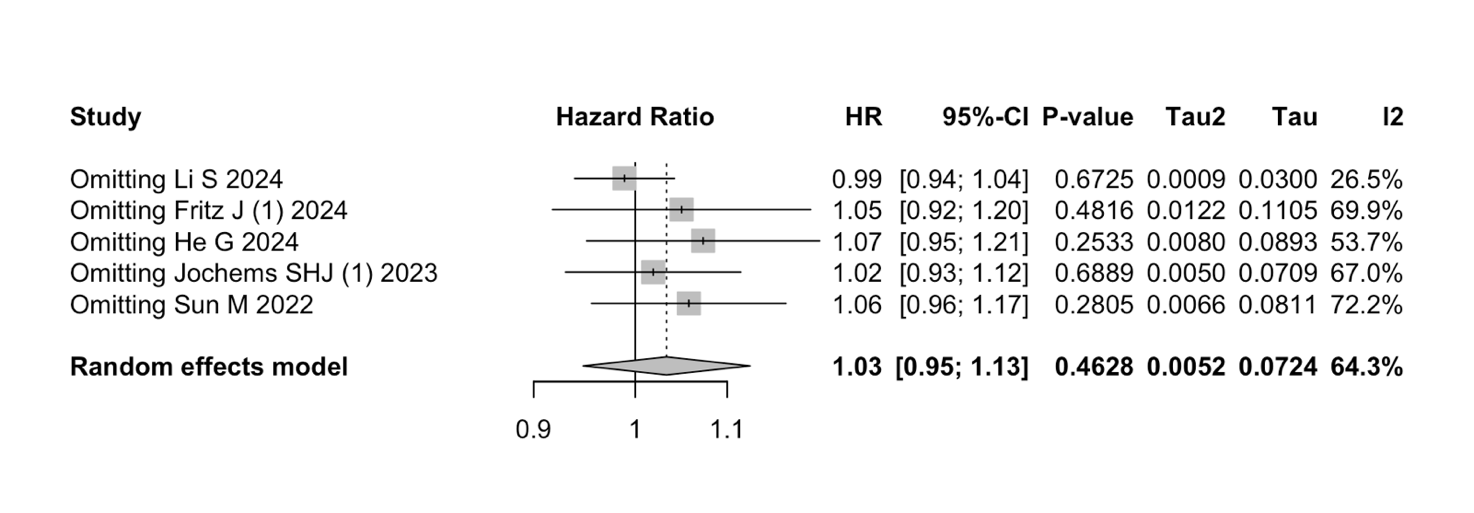


**Supplementary Figure 4.** Sensitivity analysis of the TyG index on the risk of all-cause mortality among cancer patients (categorized).


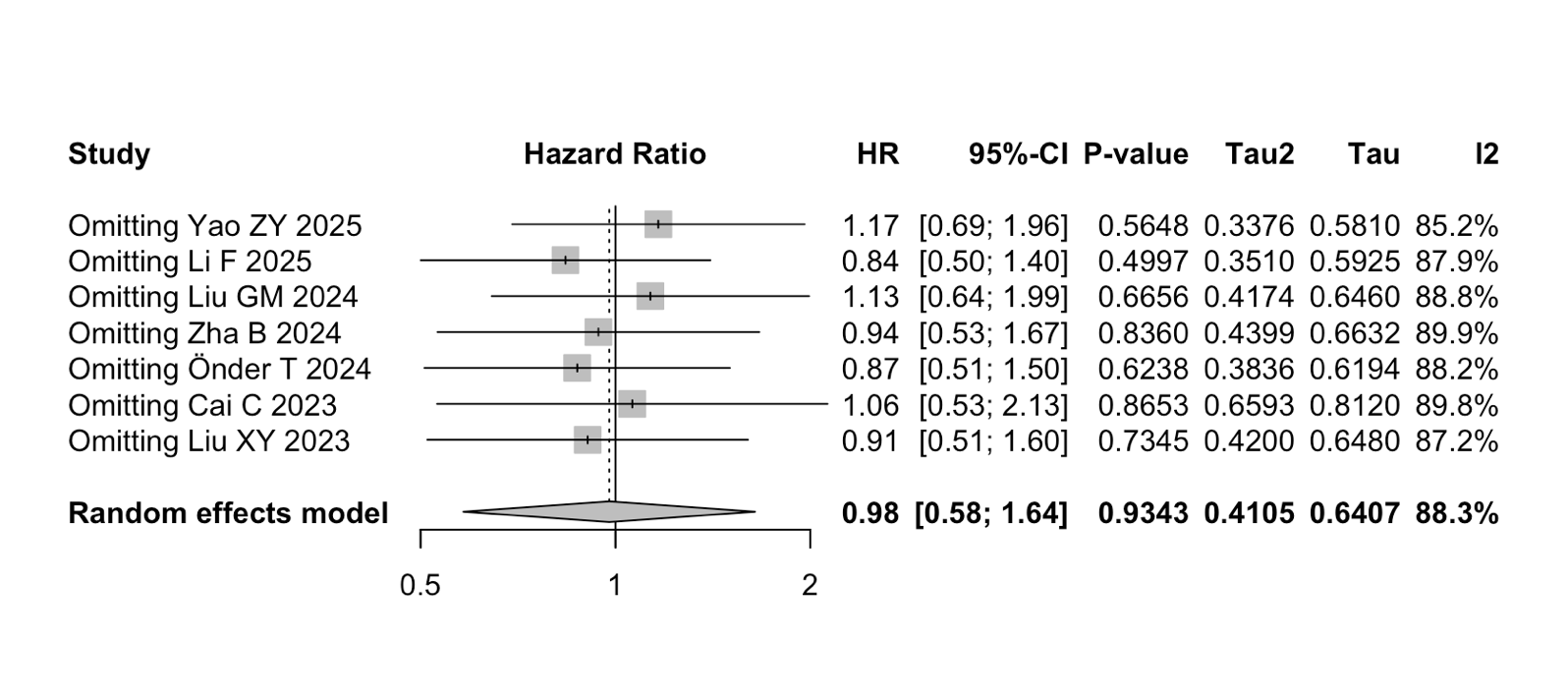

Supplement: Supplementary file 1 [file DataSheet1.docx]
