# Supplementary material for: The association of triglyceride-glucose index with cancer incidence and mortality: a systematic review and meta-analysis of cohort studies
Source: Front Endocrinol (Lausanne). 2025 Oct 24;16:1682062. doi: 10.3389/fendo.2025.1682062 (PMC12591978; doi:10.3389/fendo.2025.1682062)

**Supplementary Figures**

**Supplementary Figure 5.** Funnel plot and Egger test of the pooled estimates of cancer incidence (A. categorized; B. continuous). (A). Egger test result: t = 2.06, df = 14, p-value = 0.0584, Bias estimate: 1.9990 (SE = 0.9701); (B). Egger test result: t = 1.12, df = 15, p-value = 0.2812, Bias estimate: 0.9496 (SE = 0.8495).

A.
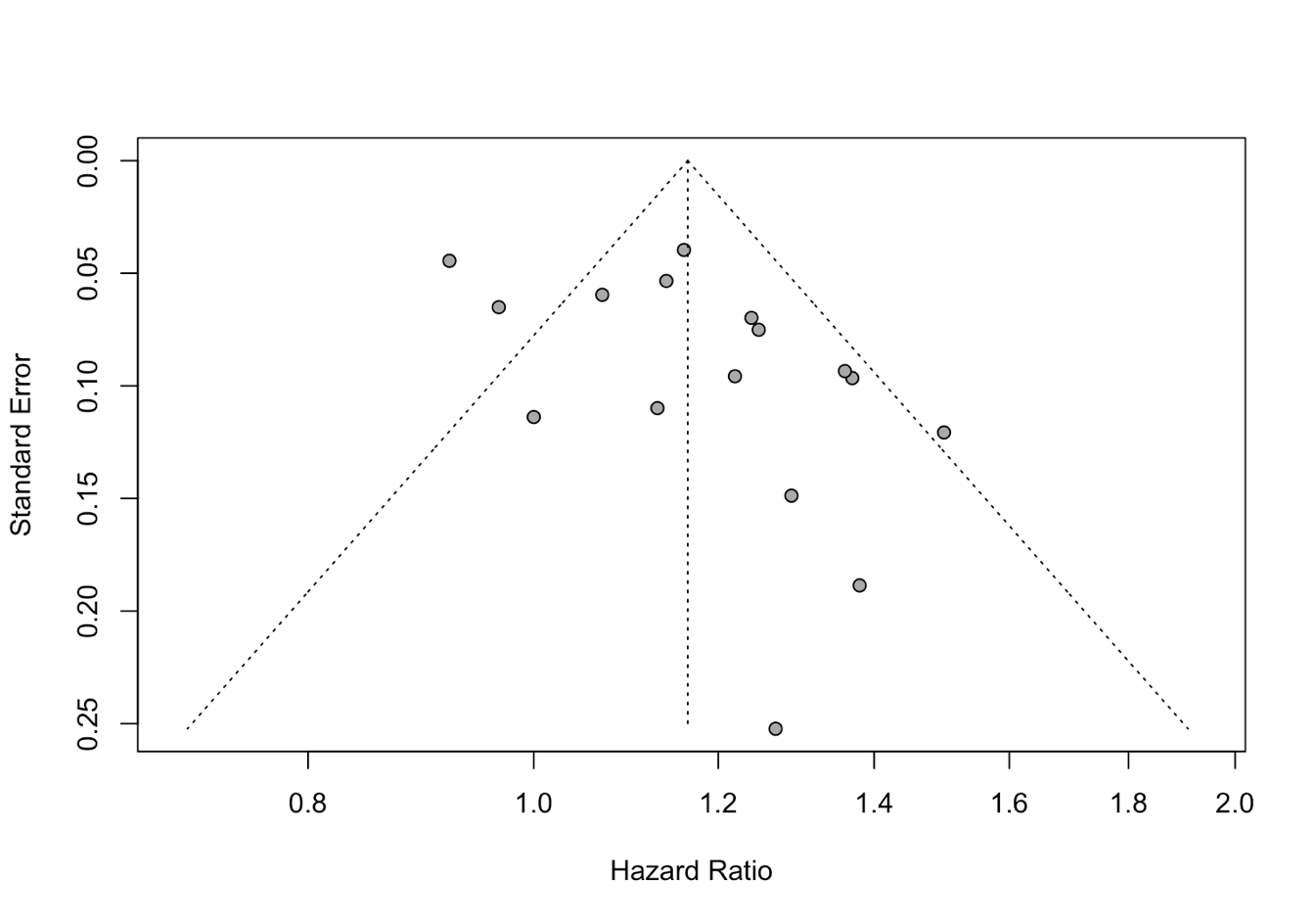


B.
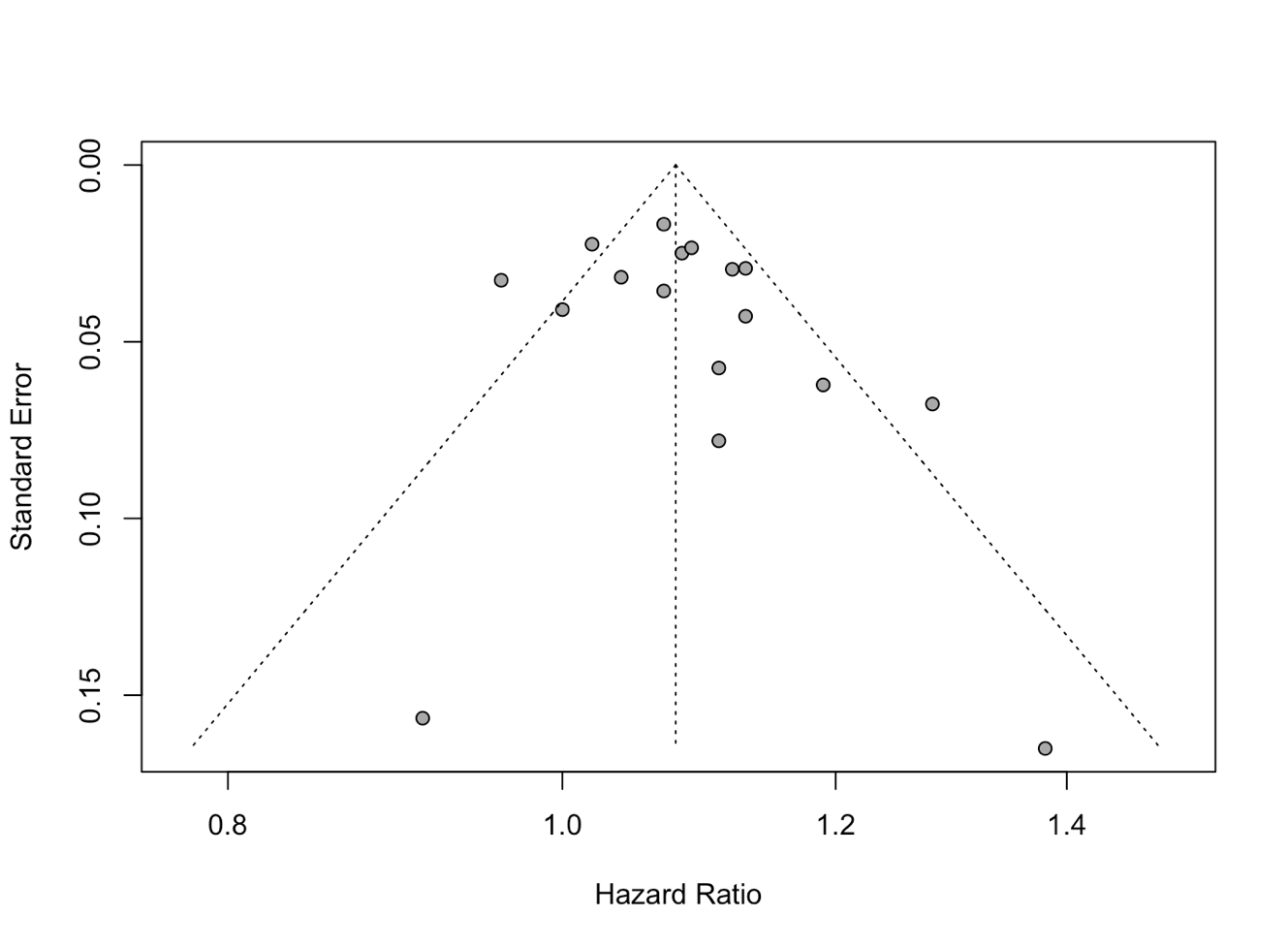


**Supplementary Figure 6.** Funnel plot and Egger test of the pooled estimates of cancer-specific mortality among cancer-free people (A. categorized; B. continuous). (A). Egger test result: t = 0.69, df = 3, p-value = 0.5376, Bias estimate: 1.1813 (SE = 1.7023); (B). Egger test result: t =1.74, df = 3, p-value = 0.1795, Bias estimate: 1.4965 (SE = 0.8579).

A.
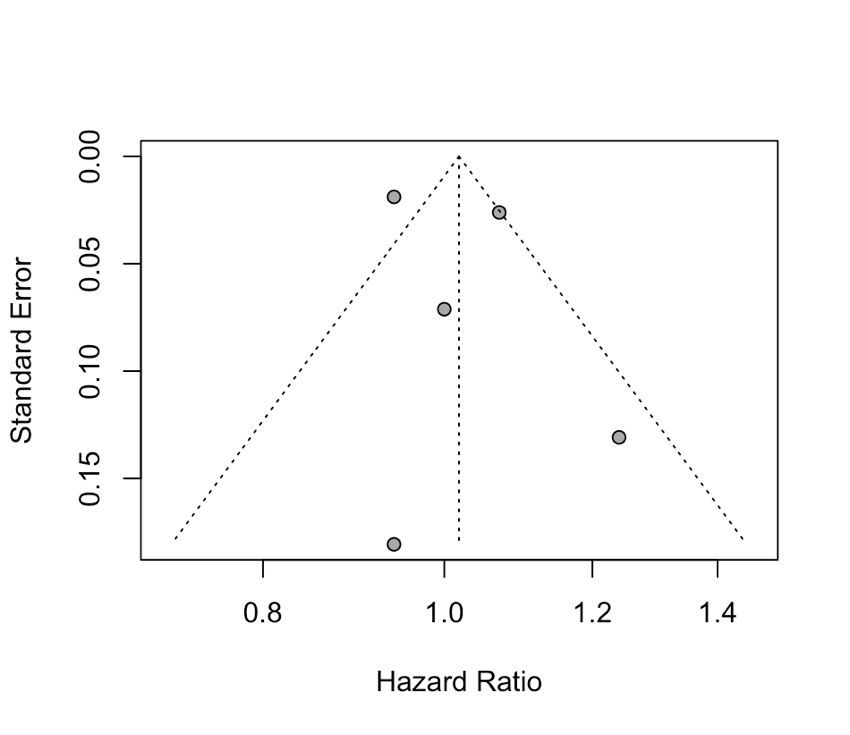


B.
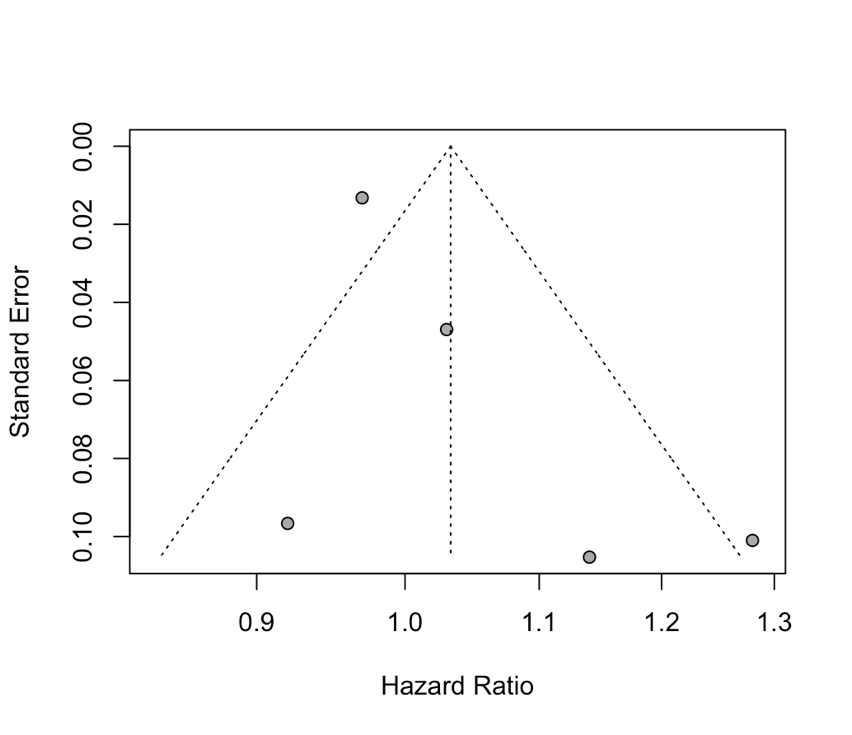


**Supplementary Figure 7.** Funnel plot and Egger test of the pooled estimates of all-cause mortality in cancer patients (categorized). Egger test result: t = 0.97, df = 5, p-value = 0.3782, Bias estimate: 2.7736 (SE = 2.8696).


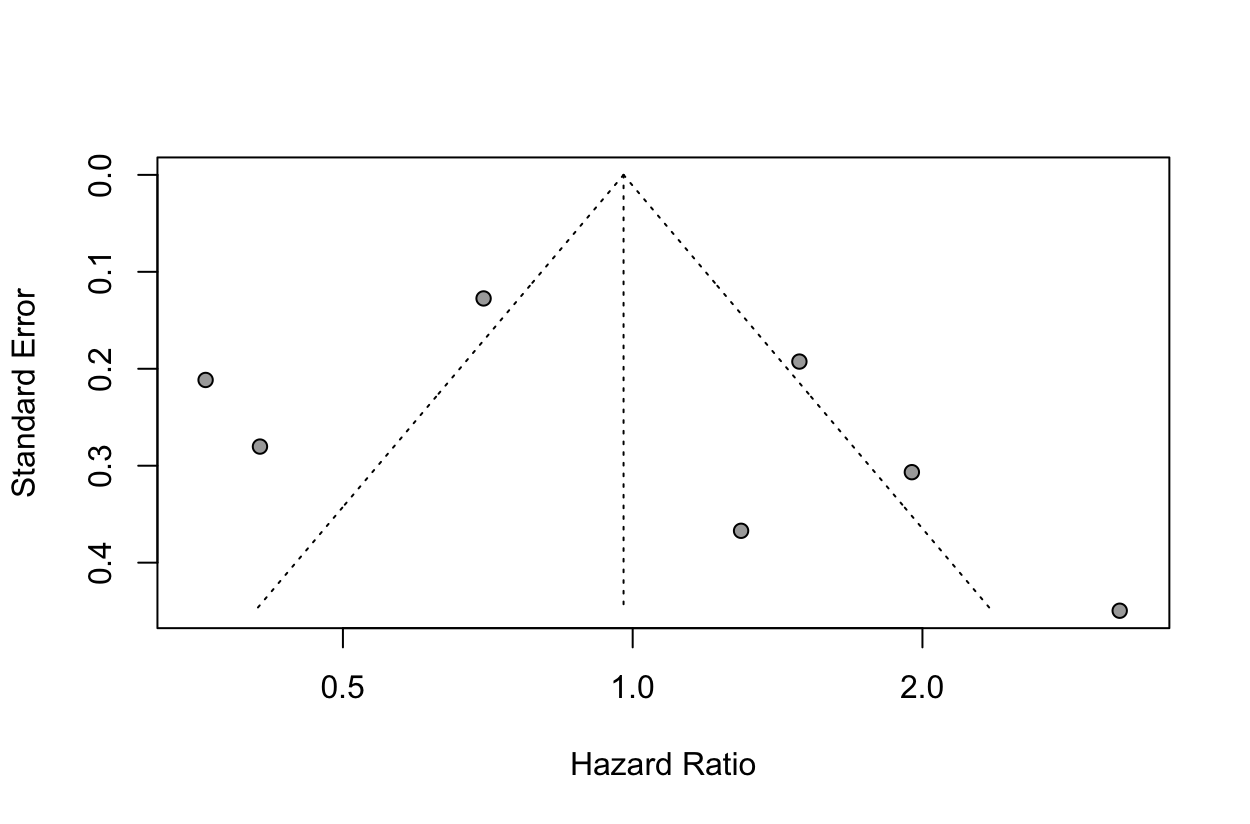


**Supplementary Figure 8.** Forest plot for the meta-analysis of the association between TyG index and the risk of cancer-related mortality among cancer-free people with the rejection of one specific study (A. categorized; B. continuous).

A.
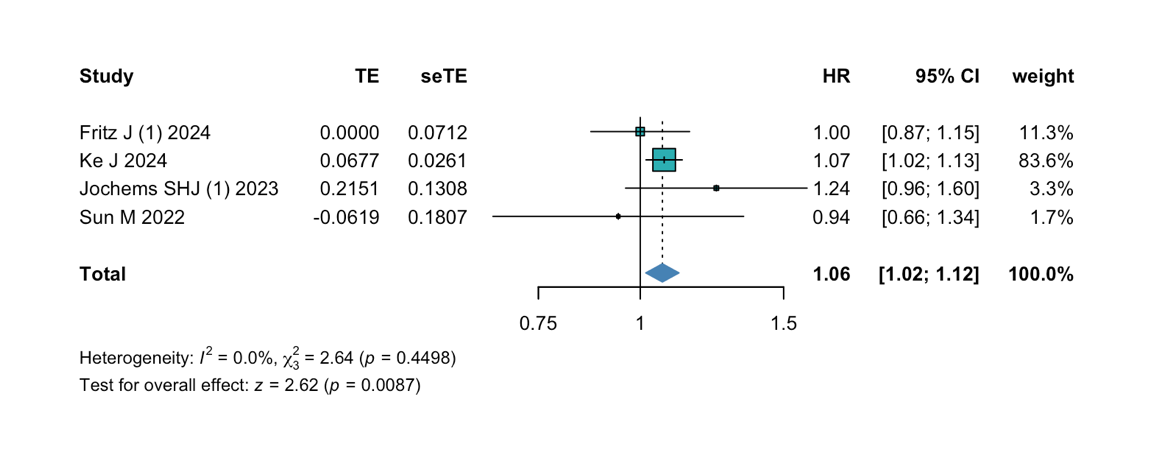


B.
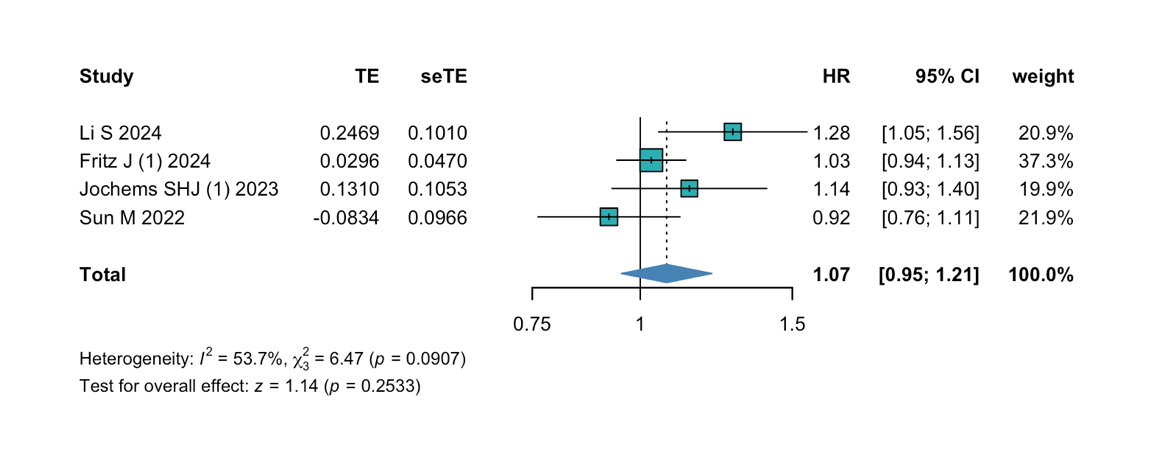

Supplement: Supplementary file 2 [file DataSheet2.docx]
