# Supplementary material for: The association of triglyceride-glucose index with cancer incidence and mortality: a systematic review and meta-analysis of cohort studies
Source: Front Endocrinol (Lausanne). 2025 Oct 24;16:1682062. doi: 10.3389/fendo.2025.1682062 (PMC12591978; doi:10.3389/fendo.2025.1682062)
Supplement: Supplementary file 3 [file DataSheet3.docx]

**Supplementary Figures**

**Supplementary Figure 9.** Sub-group analysis of the association between TyG index and the cancer-related death among cancer-free people (A. categorized; B. continuous).

A.

B.
