# Supplementary material for: The association of triglyceride-glucose index with cancer incidence and mortality: a systematic review and meta-analysis of cohort studies
Source: Front Endocrinol (Lausanne). 2025 Oct 24;16:1682062. doi: 10.3389/fendo.2025.1682062 (PMC12591978; doi:10.3389/fendo.2025.1682062)
Supplement: Supplementary file 4 [file DataSheet4.docx]

**Supplementary Tables**

**Supplementary Table 1.** Details of search strategy for PubMed, Embase and Web of Science (From database inception to July 18, 2025).

| **PubMed** | | |
| --- | --- | --- |
| Search | Query | Results |
| #1 | "TyG"[Title/Abstract] OR "triglyceride-glucose index"[Title/Abstract] OR "triacylglycerol-glucose index"[Title/Abstract] OR "triglyceride glucose index"[Title/Abstract] | 2,964 |
| #2 | "cancer*"[Title/Abstract] OR "tumor*"[Title/Abstract] OR "neoplasm*"[Title/Abstract] OR "malignan*"[Title/Abstract] OR "neoplasms"[MeSH Terms] | 5,437,316 |
| #3 | #1 AND #2 | 177 |
| **EMBASE** | | |
| Search | Query | Results |
| #1 | tyg:ab,ti OR 'triglyceride-glucose index':ab,ti OR 'triacylglycerol-glucose index':ab,ti OR 'triglyceride glucose index':ab,ti | 3,522 |
| #2 | cancer*:ab,ti OR tumor*:ab,ti OR neoplasm*:ab,ti OR 'malignant neoplasm':ab,ti OR 'neoplasm'/exp | 7,952,572 |
| #3 | #1 AND #2 | 215 |
| **Web of Science** | | |
| Search | Query | Results |
| #1 | TI= (TyG or triglyceride-glucose index or triacylglycerol-glucose index or triglyceride glucose index) OR AB= (TyG or triglyceride-glucose index or triacylglycerol-glucose index or triglyceride glucose index) | 19,699 |
| #2 | TI= (cancer* or tumor* or neoplasm* or malignan*) OR AB= (cancer* or tumor* or neoplasm* or malignan*) | 5,242,395 |
| #3 | #1 AND #2 | 979 |

**Supplementary Table 2.** PRISMA checklist.

| **Section and Topic** | **Item #** | **Checklist item** | **Location where item is reported** |
| --- | --- | --- | --- |
| **TITLE** | | |  |
| Title | 1 | Identify the report as a systematic review. | Title |
| **ABSTRACT** | | |  |
| Abstract | 2 | See the PRISMA 2020 for Abstracts checklist. | Abstract |
| **INTRODUCTION** | | |  |
| Rationale | 3 | Describe the rationale for the review in the context of existing knowledge. | Introduction |
| Objectives | 4 | Provide an explicit statement of the objective(s) or question(s) the review addresses. | Introduction |
| **METHODS** | | |  |
| Eligibility criteria | 5 | Specify the inclusion and exclusion criteria for the review and how studies were grouped for the syntheses. | Methods |
| Information sources | 6 | Specify all databases, registers, websites, organisations, reference lists and other sources searched or consulted to identify studies. Specify the date when each source was last searched or consulted. | Methods |
| Search strategy | 7 | Present the full search strategies for all databases, registers and websites, including any filters and limits used. | Methods, Supplementary Table 1 |
| Selection process | 8 | Specify the methods used to decide whether a study met the inclusion criteria of the review, including how many reviewers screened each record and each report retrieved, whether they worked independently, and if applicable, details of automation tools used in the process. | Methods |
| Data collection process | 9 | Specify the methods used to collect data from reports, including how many reviewers collected data from each report, whether they worked independently, any processes for obtaining or confirming data from study investigators, and if applicable, details of automation tools used in the process. | Methods |
| Data items | 10a | List and define all outcomes for which data were sought. Specify whether all results that were compatible with each outcome domain in each study were sought (e.g. for all measures, time points, analyses), and if not, the methods used to decide which results to collect. | Tables 1-2, Supplementary Tables 5-6 |
|  | 10b | List and define all other variables for which data were sought (e.g. participant and intervention characteristics, funding sources). Describe any assumptions made about any missing or unclear information. | Tables 1-2, Supplementary Tables 5-6 |
| Study risk of bias assessment | 11 | Specify the methods used to assess risk of bias in the included studies, including details of the tool(s) used, how many reviewers assessed each study and whether they worked independently, and if applicable, details of automation tools used in the process. | Methods |
| Effect measures | 12 | Specify for each outcome the effect measure(s) (e.g. risk ratio, mean difference) used in the synthesis or presentation of results. | Methods |
| Synthesis methods | 13a | Describe the processes used to decide which studies were eligible for each synthesis (e.g. tabulating the study intervention characteristics and comparing against the planned groups for each synthesis (item #5)). | Methods |
|  | 13b | Describe any methods required to prepare the data for presentation or synthesis, such as handling of missing summary statistics, or data conversions. | Methods |
|  | 13c | Describe any methods used to tabulate or visually display results of individual studies and syntheses. | Methods |
|  | 13d | Describe any methods used to synthesize results and provide a rationale for the choice(s). If meta-analysis was performed, describe the model(s), method(s) to identify the presence and extent of statistical heterogeneity, and software package(s) used. | Methods |
|  | 13e | Describe any methods used to explore possible causes of heterogeneity among study results (e.g. subgroup analysis, meta-regression). | Methods |
|  | 13f | Describe any sensitivity analyses conducted to assess robustness of the synthesized results. | Methods |
| Reporting bias assessment | 14 | Describe any methods used to assess risk of bias due to missing results in a synthesis (arising from reporting biases). | Methods |
| Certainty assessment | 15 | Describe any methods used to assess certainty (or confidence) in the body of evidence for an outcome. | Methods |
| **RESULTS** | | |  |
| Study selection | 16a | Describe the results of the search and selection process, from the number of records identified in the search to the number of studies included in the review, ideally using a flow diagram. | Results, Figure 1 |
|  | 16b | Cite studies that might appear to meet the inclusion criteria, but which were excluded, and explain why they were excluded. | Results, Supplementary Table 3 |
| Study characteristics | 17 | Cite each included study and present its characteristics. | Table 1, Supplementary Table 5 |
| Risk of bias in studies | 18 | Present assessments of risk of bias for each included study. | Results |
| Results of individual studies | 19 | For all outcomes, present, for each study: (a) summary statistics for each group (where appropriate) and (b) an effect estimate and its precision (e.g. confidence/credible interval), ideally using structured tables or plots. | Table 2, Supplementary Table 6 |
| Results of syntheses | 20a | For each synthesis, briefly summarise the characteristics and risk of bias among contributing studies. | Results |
|  | 20b | Present results of all statistical syntheses conducted. If meta-analysis was done, present for each the summary estimate and its precision (e.g. confidence/credible interval) and measures of statistical heterogeneity. If comparing groups, describe the direction of the effect. | Results, Figures 2-4, Supplementary Figures 8-9 |
|  | 20c | Present results of all investigations of possible causes of heterogeneity among study results. | Results |
|  | 20d | Present results of all sensitivity analyses conducted to assess the robustness of the synthesized results. | Results, Supplementary Figures 2-4 |
| Reporting biases | 21 | Present assessments of risk of bias due to missing results (arising from reporting biases) for each synthesis assessed. | Results, Supplementary Figures 5-7 |
| Certainty of evidence | 22 | Present assessments of certainty (or confidence) in the body of evidence for each outcome assessed. | Results |
| **DISCUSSION** | | |  |
| Discussion | 23a | Provide a general interpretation of the results in the context of other evidence. | Discussion |
|  | 23b | Discuss any limitations of the evidence included in the review. | Strengths and limitations |
|  | 23c | Discuss any limitations of the review processes used. | Strengths and limitations |
|  | 23d | Discuss implications of the results for practice, policy, and future research. | Discussion |
| **OTHER INFORMATION** | | |  |
| Registration and protocol | 24a | Provide registration information for the review, including register name and registration number, or state that the review was not registered. | Methods |
|  | 24b | Indicate where the review protocol can be accessed, or state that a protocol was not prepared. | Methods |
|  | 24c | Describe and explain any amendments to information provided at registration or in the protocol. | None |
| Support | 25 | Describe sources of financial or non-financial support for the review, and the role of the funders or sponsors in the review. | Funding |
| Competing interests | 26 | Declare any competing interests of review authors. | Conflict of Interest |
| Availability of data, code and other materials | 27 | Report which of the following are publicly available and where they can be found: template data collection forms; data extracted from included studies; data used for all analyses; analytic code; any other materials used in the review. | Supplementary materials |

*From:*  Page MJ, McKenzie JE, Bossuyt PM, Boutron I, Hoffmann TC, Mulrow CD, et al. The PRISMA 2020 statement: an updated guideline for reporting systematic reviews. BMJ 2021;372:n71. doi: 10.1136/bmj.n71. This work is licensed under CC BY 4.0. To view a copy of this license, visit <https://creativecommons.org/licenses/by/4.0/>

**Supplementary Table 3.** List of excluded studies, with reasons for exclusion after full text reading.

| **References** | **Reason for exclusion** |
| --- | --- |
| Association of cardiometabolic factors and insulin resistance surrogates with mortality in participants from the Korean Genome and Epidemiology Study | Deficient data of appropriate adjustment |
| Insulin resistance and inflammation mediate the association of abdominal obesity with colorectal cancer risk | On the same sample |
| A Prospective Study of Obesity, Metabolic Health, and Cancer Mortality | No independent data of TyG index |
| A Prospective Study on Metabolic Risk Factors and Gallbladder Cancer in the Metabolic Syndrome and Cancer (Me-Can) Collaborative Study | No independent data of TyG index |
| Associations of Chronic Inflammation, Insulin Resistance, and Severe Obesity With Mortality, Myocardial Infarction, Cancer, and Chronic Pulmonary Disease | No independent data of TyG index |
| Prognostic significance of glucose- lipid metabolic index in pancreatic cancer patients with diabetes mellitus | No independent data of TyG index |
| Association of the triglyceride-glucose index with the occurrence and recurrence of colorectal adenomas: a retrospective study from China | No relevant outcomes |
| An Evaluation of Metabolic, Dietetic, and Nutritional Status Reveals Impaired Nutritional Outcomes in Breast Cancer Patients | No relevant outcomes |
| Glucose metabolism disorders in patients with non-functioning adrenal adenomas - single-centre experience | No relevant outcomes |
| Metabolic Profile and Negatively Association Between Insulin Resistance and Metastatic Incidence in Indonesian Primary Invasive Breast Cancer: A Cross-Sectional Study | No relevant outcomes |
| The association between TyG and all-cause/non-cardiovascular mortality in general patients with type 2 diabetes mellitus is modified by age: results from the cohort study of NHANES 1999–2018 | No relevant outcomes |
| Triglyceride-glucose (TyG) index is a predictor of arterial stiffness, incidence of diabetes, cardiovascular disease, and all-cause and cardiovascular mortality: A longitudinal two-cohort analysis | No relevant outcomes |
| The Potential Prognostic Marker TyG Index Predicts Time to Brain Metastasis at HER2 Positive Breast Cancer | No relevant outcomes |
| TRIGLYCERIDE–GLUCOSE INDEX (TYG INDEX) IS ASSOCIATED WITH HIGHER RISK OF COLORECTAL ADENOMA IN ASYMPTOMATIC INDIVIDUALS | No relevant outcomes |
| Triglyceride Glucose Index (TyG Index) and Risk of Colorectal Adenomas | No relevant outcomes |
| A Study of Atherogenic Plasma & Triglyceride-Glucose Indices and Monocyte/HDL-C Ratios in Colon Cancer Patients | Not a cohort design |
| Assessing the use of the triglyceride-glycemic index (TyG), neutrophil-lymphocyte Ratio (NLR), and platelet-lymphocyte Ratio (PLR) in distinguishing benign and malignant tumors among patients with complaints of breast mass | Not a cohort design |
| Association between triglyceride-glucose index and gastric carcinogenesis: a health checkup cohort study | Not a cohort design |
| Association of triglyceride-glucose index with the risk of prostate cancer: a retrospective study | Not a cohort design |
| Association of nafld and insulin resistance with non metastatic bladder cancer patients: A cross‐sectional retrospective study | Not a cohort design |
| Association between triglyceride glucose index and breast cancer in 142,184 Chinese adults: findings from the REACTION study | Not a cohort design |
| Association Between Triglyceride Glucose Index and Non-Small Cell Lung Cancer Risk in Chinese Population | Not a cohort design |
| Evaluation of Triglyceride-Glucose Index Elevation as a Biomarker and Risk Factor in Laryngeal Squamous Cell Carcinoma | Not a cohort design |
| Nondiabetic patients with either subclinical Cushing's or nonfunctional adrenal incidentalomas have lower insulin sensitivity than healthy controls: clinical implications | Not a cohort design |
| Prognostic importance of systemic inflammation and insulin resistance in patients with cancer: a prospective multicenter study | Not a cohort design |
| Predictive value of triglyceride/glucose index (TyG) in predicting breast cancer in patients with breast mass | Not a cohort design |
| The Association Between Triglyceride-Glucose Index as a Marker of Insulin Resistance and the Risk of Breast Cancer | Not a cohort design |
| The relationship between papillary thyroid cancer and triglyceride/glucose index, which is an indicator of insulin resistance | Not a cohort design |
| The relationship between Triglyceride and glycose (TyG) index and the risk of gynaecologic and breast cancers | Not a cohort design |
| The Importance of Metabolic Factors in Endometrial Cancer: Evaluating the Utility of the Triglyceride-to-Glycemia Index and Triglyceride-to-High-Density Lipoprotein Ratio As Biomarkers | Not a cohort design |
| Triglyceride-glucose index is a predictor of the risk of prostate cancer: a retrospective study based on a transprostatic aspiration biopsy population | Not a cohort design |
| Triglyceride-glucose index (TyG index) and endometrial carcinoma risk: A retrospective cohort study | Not a cohort design |
| Triglyceride glucose index and Atherogenic index of plasma for predicting colorectal neoplasms in patients without cardiovascular diseases | Not a cohort design |
| Triglyceride-Glucose Index is an Independent Risk Factor for Hepatocellular Carcinoma Development in Patients with HBV-Related Liver Cirrhosis | Not a cohort design |
| Triglyceride-glucose index is a risk factor for breast cancer in China: a cross-sectional study | Not a cohort design |
| Triglyceride-glucose index and glycemic dynamics in pancreatic ductal adenocarcinoma: implications for disease progression and prognosis | Not a cohort design |

**Supplementary Table 4.** Newcastle-Ottawa quality scale for cohort study assessment.

Supplementary Table 4.1 Methodological quality of cohort studies with cancer-free people participation included in the meta-analysis^*^.

| **First author,**  **publication year**  **(reference)** | **Selection** | | | | **Comparability** | **Outcome** | | | **Total score** |
| --- | --- | --- | --- | --- | --- | --- | --- | --- | --- |
|  | **Representativeness of the exposed cohort** | **Selection of the unexposed**  **cohort** | **Ascertainment**  **of exposure** | **Outcome of interest not present at start of study** | **Control for**  **important factor or additional factor†** | **Assessment of outcome** | **Follow-up**  **long enough for outcomes**  **to occur ‡** | **Adequacy of**  **follow-up**  **of cohorts §** |  |
| Wu Z, 2025 | 1 | 1 | 1 | 1 | 2 | 1 | 1 | 1 | 9 |
| Yang C, 2024 | 1 | 1 | 1 | 1 | 1 | 1 | 1 | 1 | 8 |
| Son M, 2024 | 1 | 1 | 1 | 1 | 2 | 1 | 1 | 1 | 9 |
| Kityo A, 2024 | 1 | 1 | 1 | 1 | 1 | 1 | 1 | 1 | 8 |
| Li S, 2024 | 1 | 1 | 1 | 1 | 1 | 1 | 1 | 1 | 8 |
| Fritz J, 2024 | 1 | 1 | 1 | 1 | 1 | 1 | 1 | 1 | 8 |
| He G, 2024 | 1 | 1 | 1 | 1 | 1 | 1 | 0 | 1 | 7 |
| Ke J, 2024 | 1 | 1 | 1 | 1 | 1 | 1 | 1 | 1 | 8 |
| Jochems SHJ, 2023 | 1 | 1 | 1 | 1 | 1 | 1 | 1 | 1 | 8 |
| Liu T, 2022 | 0 | 1 | 1 | 1 | 2 | 1 | 1 | 1 | 8 |
| Sun M, 2022 | 1 | 1 | 1 | 1 | 2 | 1 | 0 | 1 | 8 |
| Wang L, 2021 | 1 | 1 | 1 | 1 | 1 | 1 | 0 | 1 | 7 |
| Okamura T, 2020 | 0 | 1 | 1 | 0 | 1 | 1 | 0 | 1 | 5 |
| Fritz J, 2020 | 1 | 1 | 1 | 1 | 1 | 1 | 1 | 1 | 8 |

* A study could be awarded a maximum of one point for each item except for the item Control for important factor or additional factor.

† A maximum of 2 points could be awarded for this item. Studies that controlled for age (important factor), sex, physical activity status, drinking, smoking, hypertension, obesity/BMI and diabetes received two points.

‡ A cohort study with a follow-up time >10 y was assigned one point.

§ A cohort study with a follow-up rate >90% was assigned one point.

Supplementary Table 4.2 Methodological quality of cohort studies with cancer-diagnosed people participation included in the meta-analysis^*^.

| **First author,**  **publication year**  **(reference)** | **Selection** | | | | **Comparability** | **Outcome** | | | **Total score** |
| --- | --- | --- | --- | --- | --- | --- | --- | --- | --- |
|  | **Representativeness of the exposed cohort** | **Selection of the unexposed**  **cohort** | **Ascertainment**  **of exposure** | **Outcome of interest not present at start of study** | **Control for**  **important factor or additional factor†** | **Assessment of outcome** | **Follow-up**  **long enough for outcomes**  **to occur ‡** | **Adequacy of**  **follow-up**  **of cohorts §** |  |
| Yao ZY, 2025 | 0 | 1 | 1 | 1 | 1 | 1 | 0 | 1 | 6 |
| Li F, 2025 | 0 | 1 | 1 | 1 | 1 | 1 | 1 | 1 | 7 |
| Liu GM, 2024 | 0 | 1 | 1 | 1 | 2 | 1 | 0 | 1 | 7 |
| Zha B, 2024 | 1 | 1 | 1 | 1 | 1 | 0 | 1 | 1 | 7 |
| Önder T, 2024 | 0 | 1 | 1 | 1 | 2 | 1 | 0 | 1 | 7 |
| Qin G, 2024 | 0 | 1 | 1 | 1 | 1 | 1 | 1 | 1 | 7 |
| Fritz J, 2024 | 1 | 1 | 1 | 1 | 1 | 1 | 1 | 1 | 8 |
| Cai C, 2023 | 0 | 1 | 1 | 1 | 2 | 1 | 0 | 1 | 7 |
| Liu XY, 2023 | 1 | 1 | 1 | 1 | 2 | 1 | 1 | 1 | 9 |
| Jochems SHJ, 2023 | 1 | 1 | 1 | 1 | 2 | 1 | 1 | 1 | 9 |

* A study could be awarded a maximum of one point for each item except for the item Control for important factor or additional factor.

† A maximum of 2 points could be awarded for this item. Studies that controlled for age (important factor), treatment, and tumor stage received two points.

‡ A cohort study with a follow-up time >5 y was assigned one point.

§ A cohort study with a follow-up rate >90% was assigned one point.
