# Supplementary material for: The association of triglyceride-glucose index with cancer incidence and mortality: a systematic review and meta-analysis of cohort studies
Source: Front Endocrinol (Lausanne). 2025 Oct 24;16:1682062. doi: 10.3389/fendo.2025.1682062 (PMC12591978; doi:10.3389/fendo.2025.1682062)
Supplement: Supplementary file 5 [file DataSheet5.docx]

**Supplementary Tables**

**Supplementary Table 5.** Details of the characteristics of included studies.

|  | The association between TyG index and cancer incidence (N = 9) | | | | | | | | | |
| --- | --- | --- | --- | --- | --- | --- | --- | --- | --- | --- |
| ID | **Author, year** | **Country** | **Participants** | **Age (SD)** | **Male (%)** | **Follow-up period** | **Outcomes** | **TyG index analysis** | **Outcome assessment** | **Reference** |
| 1 | Wu Z, 2025 | UK | 428,152 | 56.54 (8.09) | 46.1 | 13.8 Y (median FU time) | 1,759 pancreatic cancer cases | Categorized (Q4:Q1); Continuous | ICD-10 code C25.0-C25.9 | [33] |
| 2 | Yang C, 2024 | UK | 388,900 | 57.0 | 47.2 | 13 Y (median FU time) | 779 esophageal cancer cases | Categorized (Q4:Q1); Continuous | ICD-10 code C15-C19 | [18] |
| 3 | Son M, 2024 | Korea | 314,141 | Q1: 57.8 (8.5)  Q2: 59.0 (8.8)  Q3: 59.4 (8.8)  Q4: 59.1 (8.7) | Q1: 45.6  Q2: 50.8  Q3: 55.5  Q4: 63.6 | 2009-2019 | 6,112 colorectal cancer cases | Categorized (Q4:Q1) | ICD-10 code C18- C20 | [20] |
| 4 | Kityo A, 2024 | Korea | 98,800 | 53.2 (8.3) | 33.45 | 10.6 Y (median FU time) | 699 colorectal cancer cases | Continuous | ICD-10 code C18-C21 | [21] |
| 5 | Jochems SHJ, 2023 | Sweden | 68,147 | 47.3 (10.3) | 100 | 1974-2016 | 3,940 prostate cancer cases | Categorized (T3:T1); Continuous | ICD-7 code 177 | [29] |
| 6 | Liu T, 2022 | China | 93,659 | 51.44 (12.45) | 79.73 | 13.02 Y (median FU time) | 593 colorectal cancer cases | Categorized (Q4:Q1); Continuous | ICD-10 code C18-C21 | [17] |
| 7 | Wang L, 2021 | UK | 324,334 | 55.831 (8.051) | 44.176 | 9 Y (median FU time) | 1,593 lung cancer cases | Categorized (median); Continuous | ICD-10 code C34 | [30] |
| 8 | Okamura T, 2020 | Japan | 27,921 | 45.7 (10.1) | 58.86 | 4.4 Y (median FU time) | 116 colorectal cancer cases | Continuous | Gastroenterologist diagnosis | [27] |
| 9 | Fritz J, 2020 | Europe | 510,471 | 43.1 (10.6)  Q1: 39.6 (11.0)  Q2: 42.8 (10.7)  Q3: 43.6 (10.7)  Q4: 44.4 (10.2)  Q5: 44.9 (9.4) | 50.5  Q1: 32.3  Q2: 40.5  Q3: 48.7  Q4: 58.7  Q5: 72.5 | 17.2 Y (median FU time) | 16,052 obesity-related cancer cases | Categorized (Q5:Q1); Continuous | ICD-7 and ICD-10 | [7] |
|  | **The association between TyG index and cancer mortality (N = 16)** | | | | | | | | | |
| ID | **Author, year** | **Country** | **Participants** | **Age (SD)** | **Male (%)** | **Follow-up period** | **Outcomes** | **TyG index analysis** | **Endpoint identification** | **Reference** |
|  | **I. The association between TyG index and cancer mortality in cancer-free people (6)** | | | | | | | | | |
| 1 | Li S, 2024 | US | 27,642 (cancer-free) | 47.4 (19.2) | 50.2 | 10.2 Y (median FU time) | 914 cancer deaths | Continuous | ICD-10 code 019-043 | [26] |
| 2 | Fritz J, 2024 | Europe | 259,884 (cancer-free) | 43.3 (10.1)  Q1: 41.3 (10.2)  Q2: 43.1 (10.3)  Q3: 44.0 (10.0)  Q4: 44.9 (9.5) | 100 | 17.5 Y (median FU time) | 1,784 prostate cancer deaths | Categorized (Q4:Q1); Continuous | ICD-7 code 177 and/or ICD-10 code C61 | [22] |
| 3 | He G, 2024 | China | 3,524,459 (cancer-free) | 56  Q1: 55  Q2: 56  Q3: 57  Q4: 57 | 39.4  Q1: 41.9  Q2: 39.1  Q3: 37.9  Q4: 38.5 | 4.6 Y (median FU time) | 28,693 cancer deaths | Categorized (Q4:Q1); Continuous | ICD-10 code C00-C99 | [9] |
| 4 | Ke J, 2024 | UK | 374,792 (cancer-free) | 56.2 (8.1)  Q1: 54.0 (8.3)  Q2: 56.4 (8.1)  Q3: 57.2 (7.9)  Q4: 57.2 (7.8) | 46.5  Q1: 33.0  Q2: 41.7  Q3: 50.1  Q4: 61.0 | 13.95 Y (median FU time) | 13,054 cancer deaths | Categorized (Q4:Q1) | ICD-9 and ICD-10 codes | [19] |
| 5 | Jochems SHJ, 2023 | Sweden | 68,147 (cancer-free) | 47.3 (10.3) | 100 | 1974-2016 | 473 prostate cancer deaths | Categorized (T3:T1); Continuous | ICD-7 code 177 | [29] |
| 6 | Sun M, 2022 | US | 9,254 (cancer-free) | Q1: 60.53 (11.39)  Q2: 62.38 (11.54)  Q3: 62.87 (11.13)  Q4: 62.12 (10.66) | Q1: 44.7  Q2: 49.5  Q3: 49.6  Q4: 54.3 | 7.6 Y (median FU time) | 352 cancer deaths | Categorized (Q4:Q1); Continuous | ICD-10 code C00-C97 | [24] |
|  | **II. The association between TyG index and cancer mortality in cancer-diagnosed people (2)** | | | | | | | | | |
| 1 | Fritz J, 2024 | Europe | 11,760 (prostate cancer) | 68.0 (8.3)  (age at cancer diagnosis)  Q1: 68.1 (8.5)  Q2: 68.1 (8.4)  Q3: 68.3 (8.3)  Q4: 67.8 (8.1) | 100 | 5.8 Y (median FU time) (FU after date of cancer diagnosis) | 1,784 prostate cancer deaths | Categorized (Q4:Q1); Continuous | ICD-7 code 177 and/or ICD-10 code C61 | [22] |
| 2 | Jochems SHJ, 2023 | Sweden | 3,940 (prostate cancer) | Non-aggressive cancer: 67.2 (6.7)  Aggressive cancer: 70.9 (7.3)  (age at cancer diagnosis) | 100 | 1998-2016 (year of cancer diagnosis to the last FU) | 473 prostate cancer deaths | Categorized (T3:T1); Continuous | ICD-7 code 177 | [29] |
|  | **III. The association between TyG index and all-cause mortality in cancer-diagnosed people (8)** | | | | | | | | | |
| 1 | Yao ZY, 2025 | China | 300 (advanced gastric cancer treated with sintilimab combined with chemotherapy) | 18-75 | 49.33  Low TyG: 48.78  High TyG: 50.00 | 2021-2023 | - | Categorized (median) | OS | [32] |
| 2 | Li F, 2025 | China | 335 (breast cancer treated with neoadjuvant chemotherapy) | 46 | 0 | 5.42 Y (median FU time) | - | Categorized (median) | OS (the duration between the day of initial diagnosis and the last follow-up or death) | [35] |
| 3 | Liu GM, 2024 | China | 415 (hepatocellular carcinoma with hepatectomy as initial treatment) | 58.0 (11.07)  Low TyG: 57.5  High TyG: 60 | 89.6  Low TyG: 89.5  High TyG: 89.7 | 2.53 Y (median FU time) | 51 deaths | Categorized (median) | OS (the duration between the time of initial hepatectomy and the last follow-up or death) | [34] |
| 4 | Zha B, 2024 | US | 187 (gastrointestinal cancer) | 73.0 | 49.7 | 5.5 Y (median FU time) | - | Categorized (Q4:Q1) | Gastrointestinal cancer death from any reasons | [23] |
| 5 | Önder T, 2024 | Turkey | 333 (HR+/HER2- metastatic breast cancer treated with CDK4/6i plus endocrine therapy) | 56  Low TyG: 55.3 (12.3)  High TyG: 57.9 (11.7) | 0 | 2.39 Y (median FU time) | - | Categorized (median) | OS (the duration between the start of CDK4/6i treatment and the last follow-up or death from any reasons) | [16] |
| 6 | Qin G, 2024 | China | 651 (postoperative renal cell carcinoma cancer) | 56 | 63.3 | 6.29 Y (median FU time) | 106 deaths | Continuous | OS (the duration between the day of surgery and the last follow-up or death from any reasons) | [25] |
| 7 | Cai C, 2024 | China | 822 (gastric cancer treated with radical resection gastrectomy) | 64.65 (10.81)  Low TyG: 64.64 (11.48)  High TyG: 64.68 (9.88) | 73.2  Low TyG: 75.7  High TyG: 70.0 | 2014-2018 | - | Categorized (median) | OS | [31] |
| 8 | Liu XY, 2023 | China | 571 (female reproductive system cancer) | 52 (14) | 0 | 2013-2021 | - | Categorized (median); Continuous | OS (the duration between the day of admission and the last follow-up or death from any reasons) | [28] |

SD: standard deviation FU: follow-up TyG: triglyceride-glucose index ICD: International Classification of Diseases OS: overall survival CDK 4/6i: cyclin-dependent kinase 4/6 inhibitor.

**Supplementary Table 6.** Lists of estimates with 95% confidence intervals of the Triglyceride‐glucose index and cancer incidence and mortality.

| Author, year | Outcomes | Exposure category | Case/Population | Estimates | Effect metrics | Adjustments |
| --- | --- | --- | --- | --- | --- | --- |
| The association between TyG index and cancer incidence (N = 9) | | | | | | |
| Wu Z, 2025 | Pancreatic cancer | Q1 (≤ 6.72)  Q2 (6.72 ~ 7.09)  Q3 (7.09 ~ 7.49)  Q4 (≥ 7.49)  Per SD (0.57) increase |  | Ref  1.161 (0.999-1.349)  1.234 (1.066-1.429)  1.249 (1.078-1.447)  1.083 (1.031-1.137) | HR | Age, sex, income level, ethnicity, qualification, employment status, physical activity, hypertension, diabetes, pancreatitis history, smoking, drinking, meat intake, fruit intake, vegetable intake, glycated hemoglobin, TC, HDL-C, LDL-C, IGF-1 and Townsend deprivation index |
| Yang C, 2024 | Esophageal cancer | Q1 (≤ 8.5)  Q2 (8.5 ~ 8.7)  Q3 (8.7 ~ 8.9)  Q4 (≥ 8.9)  Per SD increase |  | Ref  0.97 (0.77-1.22)  1.04 (0.84-1.29)  1.13 (0.91-1.40)  1.07 (1.00-1.15) | HR | Age, sex, ethnicity, Townsend deprivation index, Metabolic Equivalent of Task, smoking, drinking, diabetes, hypertension, insulin, fasting time and diet score |
|  | Esophageal adenocarcinoma | Q1 (≤ 8.5)  Q2 (8.5 ~ 8.7)  Q3 (8.7 ~ 8.9)  Q4 (≥ 8.9)  Per SD increase |  | Ref  1.21 (0.88-1.65)  1.40 (1.05-1.89)  1.54 (1.15-2.06)  1.16 (1.07-1.26) |  |  |
|  | Esophageal squamous cell carcinoma | Q1 (≤ 8.5)  Q2 (8.5 ~ 8.7)  Q3 (8.7 ~ 8.9)  Q4 (≥ 8.9)  Per SD increase |  | Ref  0.67 (0.46-0.97)  0.57 (0.39-0.85)  0.55 (0.36-0.82)  0.80 (0.67-0.95) |  |  |
| Son M, 2024 | Colorectal cancer | Q1  Q2  Q3  Q4 | 1266/78,613  1468/77,345  1606/78,955  1772/79,228 | Ref  1.08 (1.00-1.16)  1.10 (1.02-1.19)  1.16 (1.07-1.25) | HR | Age, sex, income level, residence, hypertension, diabetes, dyslipidemia, Charlson comorbidity index, BMI, hemoglobin, GFR, smoking, drinking and regular exercise status |
|  | Colon cancer | Q1  Q2  Q3  Q4 |  | Ref  1.05 (0.95-1.16)  1.09 (0.99-1.20)  1.12 (1.01-1.24) |  |  |
|  | Rectal cancer | Q1  Q2  Q3  Q4 |  | Ref  1.08 (0.93-1.26)  1.22 (1.05-1.41)  1.32 (1.14-1.54) |  |  |
| Kityo A, 2024 | Colorectal cancer | Per 1 increase | 699/98,800 | 1.28 (1.12-1.46) | HR | Age, sex, educational level, monthly income, smoking, drinking, regular exercise status, BMI, fruit and vegetable intake, and total red meat intake |
|  | Colon cancer | Per 1 increase | 422/98,800 | 1.29 (1.10-1.54) |  |  |
|  | Rectal cancer | Per 1 increase | 277/98,800 | 1.24 (1.01-1.52) |  |  |
| Jochems SHJ, 2023 | Prostate cancer | T1  T2  T3  Per SD (0.5) increase | 1165/18,927  1114/18,984  1046/18,986  3325/56,897 | Ref  0.96 (0.88-1.04)  0.92 (0.84-1.00)  0.96 (0.88-1.00) | HR | Age, year of birth, cohort, diabetes, country of birth, educational level, BMI and smoking |
| Liu T, 2022 | Colorectal cancer | Q1 (≤ 8.19)  Q2 (8.19 ~ 8.58)  Q3 (8.58 ~ 9.06)  Q4 (≥ 9.06)  Per 1 increase |  | Ref  1.13 (0.88-1.45)  1.36 (1.06-1.76)  1.50 (1.19-1.91)  1.19 (1.05-1.34) | HR | Age, sex, family income, educational level, marital status, WC, TC, smoking, drinking, physical activity, sedentary lifestyle, tea consumption, salt intake, high-fat diet, hypertension, family history of cancer, and diabetes |
| Wang L, 2021 | Lung cancer | Low TyG (< 8.639)  High TyG (≥ 8.639)  Per SD (0.541) increase |  | Ref  0.966 (0.850-1.097)  0.911 (0.640-1.182) | HR | Age, sex, region, Townsend deprivation score, smoking, drinking, BMI, WHR, hypertension, fasting time, TC, LDL-C, HDL-C and glycated hemoglobin |
| Okamura T, 2020 | Colorectal cancer | Per 1 increase | 116/27,944 | 1.38 (1.00-1.91) | HR | Age, sex, BMI, smoking, drinking, exercise, systolic blood pressure and serum creatinine |
| Fritz J, 2020 | Esophageal adenocarcinoma | Q1 (< 8.1)  Q2 (8.1 ~ 8.4)  Q3 (8.4 ~ 8.7)  Q4 (8.7 ~ 9.1)  Q5 (> 9.1)  Per SD (0.60) increase | 185/510,471 | Ref  0.97 (0.58-1.62)  1.09 (0.66-1.80)  1.25 (0.77-2.04)  1.27 (0.77-2.07)  1.11 (0.95-1.29) | HR | Age, sex, smoking, fasting status, cohort, birth and BMI |
|  | Colon cancer | Q1 (< 8.1)  Q2 (8.1 ~ 8.4)  Q3 (8.4 ~ 8.7)  Q4 (8.7 ~ 9.1)  Q5 (> 9.1)  Per SD (0.60) increase | 4032/510,471 | Ref  0.98 (0.88-1.10)  1.07 (0.96-1.19)  1.16 (1.04-1.29)  1.14 (1.03-1.27)  1.07 (1.03-1.10) |  |  |
|  | Rectal cancer | Q1 (< 8.1)  Q2 (8.1 ~ 8.4)  Q3 (8.4 ~ 8.7)  Q4 (8.7 ~ 9.1)  Q5 (> 9.1)  Per SD (0.60) increase | 2430/510,471 | Ref  1.04 (0.90-1.19)  1.12 (0.98-1.28)  1.13 (0.99-1.30)  1.24 (1.08-1.42)  1.09 (1.04-1.14) |  |  |
|  | Liver cancer | Q1 (< 8.1)  Q2 (8.1 ~ 8.4)  Q3 (8.4 ~ 8.7)  Q4 (8.7 ~ 9.1)  Q5 (> 9.1)  Per SD (0.60) increase | 561/510,471 | Ref  1.13 (0.83-1.54)  1.11 (0.82-1.50)  1.01 (0.74-1.37)  1.29 (0.96-1.72)  1.13 (1.04-1.23) |  |  |
|  | Gallbladder cancer | Q1 (< 8.1)  Q2 (8.1 ~ 8.4)  Q3 (8.4 ~ 8.7)  Q4 (8.7 ~ 9.1)  Q5 (> 9.1)  Per SD (0.60) increase | 364/510,471 | Ref  1.23 (0.84-1.81)  1.21 (0.83-1.77)  1.15 (0.79-1.68)  1.38 (0.95-1.99)  1.11 (0.99-1.24) |  |  |
|  | Pancreatic cancer | Q1 (< 8.1)  Q2 (8.1 ~ 8.4)  Q3 (8.4 ~ 8.7)  Q4 (8.7 ~ 9.1)  Q5 (> 9.1)  Per SD (0.60) increase | 1368/510,471 | Ref  1.19 (0.98-1.44)  1.20 (1.00-1.46)  1.27 (1.05-1.53)  1.37 (1.13-1.65)  1.12 (1.06-1.19) |  |  |
|  | Pancreatic cancer (male) | Q1 (< 8.1)  Q2 (8.1 ~ 8.4)  Q3 (8.4 ~ 8.7)  Q4 (8.7 ~ 9.1)  Q5 (> 9.1)  Per SD (0.60) increase | 776/510,471 | Ref  1.12 (0.88-1.43)  1.19 (0.94-1.52)  1.11 (0.87-1.42)  1.25 (0.98-1.59)  1.08 (1.00-1.16) |  |  |
|  | Pancreatic cancer (female) | Q1 (< 8.1)  Q2 (8.1 ~ 8.4)  Q3 (8.4 ~ 8.7)  Q4 (8.7 ~ 9.1)  Q5 (> 9.1)  Per SD (0.60) increase | 592/510,471 | Ref  1.32 (0.96-1.81)  1.24 (0.90-1.70)  1.54 (1.13-2.08)  1.58 (1.16-2.14)  1.19 (1.09-1.31) |  |  |
|  | Breast cancer (postmenopausal) | Q1 (< 8.1)  Q2 (8.1 ~ 8.4)  Q3 (8.4 ~ 8.7)  Q4 (8.7 ~ 9.1)  Q5 (> 9.1)  Per SD (0.60) increase | 3427/510,471 | Ref  1.04 (0.92-1.18)  1.08 (0.96-1.22)  1.07 (0.95-1.20)  1.07 (0.95-1.20)  1.02 (0.98-1.07) |  |  |
|  | Endometrium cancer | Q1 (< 8.1)  Q2 (8.1 ~ 8.4)  Q3 (8.4 ~ 8.7)  Q4 (8.7 ~ 9.1)  Q5 (> 9.1)  Per SD (0.60) increase | 1417/510,471 | Ref  1.27 (1.05-1.54)  1.07 (0.88-1.29)  1.28 (1.06-1.54)  1.22 (1.01-1.47)  1.04 (0.98-1.11) |  |  |
|  | Ovary cancer | Q1 (< 8.1)  Q2 (8.1 ~ 8.4)  Q3 (8.4 ~ 8.7)  Q4 (8.7 ~ 9.1)  Q5 (> 9.1)  Per SD (0.60) increase | 921/510,471 | Ref  1.03 (0.83-1.27)  0.89 (0.72-1.11)  1.04 (0.84-1.29)  1.00 (0.80-1.25)  1.00 (0.92-1.08) |  |  |
|  | Kidney renal cell cancer | Q1 (< 8.1)  Q2 (8.1 ~ 8.4)  Q3 (8.4 ~ 8.7)  Q4 (8.7 ~ 9.1)  Q5 (> 9.1)  Per SD (0.60) increase | 1347/510,471 | Ref  1.06 (0.87-1.28)  1.02 (0.84-1.23)  1.18 (0.98-1.42)  1.36 (1.13-1.63)  1.13 (1.07-1.20) |  |  |
|  | Digestive organs cancer Combined | Q1 (< 8.1)  Q2 (8.1 ~ 8.4)  Q3 (8.4 ~ 8.7)  Q4 (8.7 ~ 9.1)  Q5 (> 9.1)  Per SD (0.60) increase | 8940/510,471 | Ref  1.04 (0.97-1.12)  1.11 (1.03-1.19)  1.16 (1.08-1.24)  1.22 (1.14-1.31)  1.09 (1.06-1.11) |  |  |
|  | Endometrium, ovary and breast (postmenopausal) cancer Combined | Q1 (< 8.1)  Q2 (8.1 ~ 8.4)  Q3 (8.4 ~ 8.7)  Q4 (8.7 ~ 9.1)  Q5 (> 9.1)  Per SD (0.60) increase | 5765/510,471 | Ref  1.09 (1.00-1.20)  1.05 (0.96-1.15)  1.11 (1.02-1.22)  1.09 (1.00-1.20)  1.03 (0.99-1.06) |  |  |
| The association between TyG index and cancer mortality in cancer-free people (6) | | | | | | |
| Li S, 2024 | Cancer death | Per SD (0.69) increase  Per 1 increase |  | 1.18 (1.03-1.36)  1.28 (1.05-1.56) | HR | Age, sex, ethnicity, BMI, smoking, drinking, systolic blood pressure, diabetes, ASCVD, COPD, chronic heart failure, chronic renal disease, cancer, TC, HDL-C, LDL-C, albumin, eGFR, statin use, and insulin or antihyperglycemic drugs |
| Fritz J, 2024 | Prostate cancer death | Q1  Q2  Q3  Q4  Per SD (0.6) increase | 1784/259,884 | Ref  0.99 (0.86-1.13)  1.05 (0.92-1.21)  1.00 (0.87-1.15)  1.03 (0.94-1.13) | HR | Age at study entry, BMI, smoking, fasting status, cohort and birth |
| He G, 2024 | Cancer death | Q1 (< 8.41)  Q2 (8.41 ~ 8.76)  Q3 (8.76 ~ 9.16)  Q4 (≥ 9.16)  Per 1 increase | 7634/880,296  7399/883,137  6944/879,621  6716/881,405  28693/3,524,459 | Ref  0.99 (0.96-1.03)  0.97 (0.94-1.01)  0.94 (0.91-0.98)  0.97 (0.94-0.99) | HR | Age, sex, educational level, annual household income, smoking, drinking, central obesity, systolic blood pressure, LDL-C, HDL-C, COPD, cancer, diabetes, and lipid-lowering drug use |
| Ke J, 2024 | Cancer death | Q1 (< 8.31)  Q2 (8.31 ~ 8.67)  Q3 (8.68 ~ 9.07)  Q4 (≥ 9.08) | 2998/93,478  2448/92,728  3616/94,421  3992/94,165 | 1.06 (1.01-1.12)  Ref  1.05 (1.00-1.11)  1.07 (1.02-1.13) | HR | Age, sex, smoking, drinking, BMI, hypertension and CRP |
| Jochems SHJ, 2023 | Prostate cancer death | T1  T2  T3  Per SD increase | 121/18,927  139/18,984  147/18,986  407/56,897 | Ref  1.13 (0.89-1.45)  1.24 (0.97-1.62)  1.14 (0.92-1.39) | HR | Age, year of birth, cohort, diabetes, country of birth, educational level, BMI and smoking |
| Sun M, 2022 | Cancer death | Q1 (≤ 8.32)  Q2 (8.32 ~ 8.72)  Q3 (8.72 ~ 9.16)  Q4 (> 9.16)  Per 1 increase | 71/2,306  95/2,330  88/2,318  98/2,300  352/9,254 | Ref  1.08 (0.78-1.49)  1.05 (0.75-1.46)  0.94 (0.66-1.34)  0.92 (0.76-1.11) | HR | Age, sex, ethnicity, educational level, income level, BMI, smoking, drinking, physical activity, hypertension, diabetes, CVD, COPD, liver diseases hypoglycemic drug use, lipid-lowering drug use, LDL and eGFR |
| The association between TyG index and cancer mortality in cancer-diagnosed people (2) | | | | | | |
| Fritz J, 2024 | Prostate cancer death | Q1  Q2  Q3  Q4  Per SD (0.6) increase | 1784/11,760 | Ref  1.03 (0.90-1.18)  1.15 (1.00-1.32)  1.17 (1.01-1.35)  1.11 (1.01–1.22) | HR | Age at diagnosis, BMI, smoking, fasting status, cohort and birth |
| Jochems SHJ, 2023 | Prostate cancer death | T1  T2  T3  Per SD increase | 102/1,022  125/1,042  123/1,028  350/3,092 | Ref  1.25 (0.95-1.65)  1.38 (1.04-1.82)  1.24 (1.00-1.55) | HR | Age at diagnosis, time since baseline, diabetes, country of birth, BMI, smoking, education at the time of diagnosis, cohort, birth, Charlson comorbidity index, primary treatment for prostate cancer and prostate cancer risk category |
| The association between TyG index and all-cause mortality in cancer-diagnosed people (8) | | | | | | |
| Yao ZY, 2025 | Advanced gastric cancer treated with sintilimab combined with chemotherapy death | Low TyG (< 1.79)  High TyG (≥ 1.79) | NA | Ref  0.36 (0.24-0.55) | HR | Eastern Cooperative Oncology group and PD-L1 expression |
| Li F, 2025 | Breast cancer treated with neoadjuvant chemotherapy death | Low TyG (< 8.01)  High TyG (≥ 8.01) | NA | Ref  3.206 (1.328-7.737) | HR | Age, T stage, N stage and TyG-BMI |
| Liu GM, 2024 | Hepatocellular carcinoma with hepatectomy as initial treatment death | Low TyG (< 6.58)  High TyG (≥ 6.58) | NA | 2.43 (1.39-4.24)  Ref | HR | TNM stage, AFP, tumor size, tumor number, tumor capsule, vascular invasion, tumor grade, major resuction, surgical approach, TG-HDL-c and TyG-BMI |
| Zha B, 2024 | Gastrointestinal cancer death | Q1  Q2  Q3  Q4 | NA | Ref  1.664 (0.785-3.526)  1.413 (0.649-3.079)  1.296 (0.631-2.661) | HR | Age, sex, ethnicity, educational level, smoking and drinking |
| Önder T, 2024 | HR+/HER2- metastatic breast cancer treated with CDK4/6i plus endocrine therapy death | Low TyG (< 8.43)  High TyG (≥ 8.43) | NA | 0.513 (0.281-0.936)  Ref | HR | Tumor grade, Ki67 index, progesterone receptor, CDK4/6i, Eastern Cooperative Oncology Group, visceral disease |
| Qin G, 2024 | Postoperative renal cell carcinoma death | Per SD (4.31) increase | NA | 1.729 (1.150-2.60) | HR | Age, HDL-C, diabetes, necrosis, tumorthrombus, tumor size and tumor stage |
| Cai C, 2023 | Gastric cancer death | Low TyG (≤ 1.4)  High TyG (> 1.4) | NA | Ref  0.70 (0.54-0.89) | HR | Age, hemoglobin, albumin, tumor site, laparoscopic surgery, surgical procedure, type of resuction, combined resuction, T stage, N stage, TNM stage, nutritional risk screening, histological type and postoperative complications |
| Liu XY, 2023 | Female reproductive system cancer death | Low TyG (≤ 4.62)  High TyG (> 4.62)  Per 1 increase | NA | Ref  1.49 (1.02-2.17)  1.11 (0.95-1.30) | HR | Age, tumor stage, surgery, chemotherapy, radiotherapy and albumin |

SD: standard deviation TyG: triglyceride-glucose index Ref: reference HR: hazard ratio OS: overall survival NA: not applied TC: total cholesterol HDL-C: high-density lipoprotein cholesterol LDL-C: low-density lipoprotein cholesterol IGF-1: insulin-like growth factor 1 BMI: body mass index GFR: glomerular filtration rate WC: waist circumference WHR: waist-to-hip ratio ASCVD: atherosclerotic cardiovascular disease COPD: chronic obstructive pulmonary disease CRP: C-reactive protein CVD: cardiovascular disease PD-L1: programmed cell death protein ligand 1 AFP: alpha-fetoprotein TG-HDL-c: total triglyceride/high-density lipoprotein cholesterol TyG-BMI: triglyceride-glucose body mass index TNM: Tumor-Node-Metastasis stage CDK 4/6i: cyclin-dependent kinase 4/6 inhibitor.
